# RNA binding protein with multiple splicing (RBPMS) promotes contractile phenotype splicing in human embryonic stem cell–derived vascular smooth muscle cells

**DOI:** 10.1093/cvr/cvae198

**Published:** 2024-09-09

**Authors:** Aishwarya G Jacob, Ilias Moutsopoulos, Alex Petchey, Rafael Kollyfas, Vincent R Knight-Schrijver, Irina Mohorianu, Sanjay Sinha, Christopher W J Smith

**Affiliations:** Department of Biochemistry, University of Cambridge, Cambridge CB2 1QW, UK; MRC-Wellcome Cambridge Stem Cell Institute, Cambridge CB2 0AW, UK; MRC-Wellcome Cambridge Stem Cell Institute, Cambridge CB2 0AW, UK; MRC-Wellcome Cambridge Stem Cell Institute, Cambridge CB2 0AW, UK; MRC-Wellcome Cambridge Stem Cell Institute, Cambridge CB2 0AW, UK; MRC-Wellcome Cambridge Stem Cell Institute, Cambridge CB2 0AW, UK; MRC-Wellcome Cambridge Stem Cell Institute, Cambridge CB2 0AW, UK; MRC-Wellcome Cambridge Stem Cell Institute, Cambridge CB2 0AW, UK; Department of Biochemistry, University of Cambridge, Cambridge CB2 1QW, UK

**Keywords:** Alternative splicing, Vascular smooth muscle, RNA-binding proteins

## Abstract

**Aims:**

Differentiated vascular smooth muscle cells (VSMCs) express a unique network of mRNA isoforms via smooth muscle–specific alternative pre-mRNA splicing (SM-AS) in functionally critical genes, including those comprising the contractile machinery. We previously described RNA Binding Protein with Multiple Splicing (RBPMS) as a potent driver of differentiated SM-AS in the rat PAC1 VSMC cell line. What is unknown is how RBPMS affects VSMC phenotype and behaviour. Here, we aimed to dissect the role of RBPMS in SM-AS in human cells and determine the impact on VSMC phenotypic properties.

**Methods and results:**

We used human embryonic stem cell–derived VSMCs (hESC-VSMCs) as our platform. hESC-VSMCs are inherently immature, and we found that they display only partially differentiated SM-AS patterns while RBPMS protein levels are low. We found that RBPMS over-expression induces SM-AS patterns in hESC-VSMCs akin to the contractile tissue VSMC splicing patterns. We present *in silico* and experimental findings that support RBPMS’ splicing activity as mediated through direct binding and via functional cooperativity with splicing factor RBFOX2 on a significant subset of targets. We also demonstrate that RBPMS can alter the motility and the proliferative properties of hESC-VSMCs to mimic a more differentiated state.

**Conclusion:**

Overall, this study emphasizes a critical role for RBPMS in establishing the contractile phenotype splicing programme of human VSMCs.

## Graphical Abstract

**Graphical Abstract:**
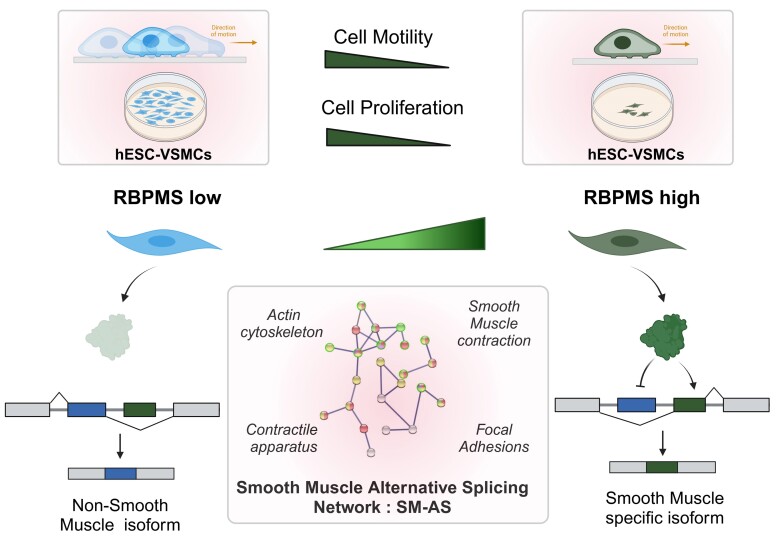

**Time of primary review: 24 days**

## 1. Introduction

Vascular smooth muscle cells (VSMCs) constitute the major architectural component of the large arteries. In healthy vessels, VSMCs have a mature or differentiated, contractile phenotype, functioning as effectors of vascular tone and mediators of tonic contraction to ensure effective blood flow (vasoconstriction and vasodilation).^1,2^ These cells express several markers that are reflective of these properties including smooth muscle myosin heavy chain (SM-MHC or MYH11), Calponin1 (CNN1), and TAGLN.^2–4^ Notably, VSMCs are phenotypically plastic, and in response to vessel wall injury and in several cardiovascular conditions including atherosclerosis, restenosis, and hypertension, they dedifferentiate into more mesenchymal states that are associated with highly proliferative and synthetic characteristics.^1–3,5,6^ This phenotypic switch is accompanied by major changes in the cellular transcriptome including loss of contractile marker expression. However, current definitions of the molecular networks defining VSMC phenotypes focus on relative mRNA abundance profiles of marker genes,^2,7^ leaving the post-transcriptional component of the VSMC transcriptome largely unexplored.

Alternative pre-mRNA splicing (AS) is a tightly regulated, pervasive post-transcriptional process that facilitates the generation of multiple, often functionally distinct transcript isoforms from the same gene.^8–10^ Previously, we showed that AS is a major component that defines arterial tissue VSMC transcriptomes and uncovered a network of mRNA isoforms expressed in contractile, mature VSMCs—the smooth muscle–specific AS (SM-AS) programme.^11,12^ This includes well-known markers of advanced VSMC maturity such as heavy-Caldesmon (CALD1)^13^ and meta-Vinculin (VCL).^14,15^ The master transcriptional regulator MYOCD also shows VSMC-specific splicing by including an alternative exon 2a producing a protein with better SRF co-activation than the skipped isoform.^16–19^ SM-AS is lost during phenotype switching alongside the contractile marker network expression.^11,12^

AS regulation is an intricate and complex process that is connected to the expression, localization, and activity of sequence-specific regulatory RNA-binding proteins (RBPs). Numerous RBPs including QKI and members of the CELF, RBFOX, and MBNL protein families have been described with roles in the cardiovascular system—both developmentally and in the context of disease, where they control several post-transcriptional processes including splicing.^20–33^ In VSMCs, a handful of RBPs including SRSF1,^34^ QKI,^18^ HuR,^35^ hnRNPA2B1,^36^ and PTBP1^11^ have been described as targeting key regulators of VSMC phenotype and transcription in the context of phenotype switching and diseases such as hypertension.^37^ The RNA Binding Protein with Multiple Splicing (RBPMS) family of genes—RBPMS and RBPMS2—are now being increasingly appreciated as regulators of smooth muscle and cardiac post-transcriptional programmes.^38–44^ Recent studies showed RBPMS as an essential gene whose homozygous deletion leads to neo-natal lethality due to under-developed myocardium with contractile defects, potentially resulting from altered splicing patterns in key functional genes.^39,40^

We previously described RBPMS as a critical regulator of SM-AS in phenotype switching of rat PAC1 VSMCs affecting nearly 20% of the SM-AS network.^12^ However, it is not clear how RBPMS impacts vascular function and VSMC phenotype as a splicing regulator in human VSMCs. Here, we use human embryonic stem cell–derived VSMCs (hESC-VSMCs) to test the hypothesis that RBPMS plays a critical role in mature, adult VSMCs, and the SM-AS network underlies differentiated or contractile VSMC phenotypic behaviour. These cells represent a foetal/immature VSMC state^45–47^ expressing only basal-level SM-AS and no detectable endogenous RBPMS. We show, using over-expression, that RBPMS is a potent driver of SM-AS akin to tissue VSMCs modulating the actin cytoskeleton, focal adhesion, and contractile machinery. We show that RBPMS functionally interacts with and requires RBFOX2, at a subset of these targets, to mediate SM-AS. Finally, we describe how RBPMS over-expressing hESC-VSMCs show lowered motility and proliferation in a manner similar to mature, differentiated VSMCs. Overall, we make a case for RBPMS as a driver of AS events associated with mature, contractile tissue VSMCs.

## 2. Methods

### 2.1 VSMC differentiation

VSMCs were differentiated from H9-rTTA hESCs^48–50^ (WiCell) via the neural crest (NC) or the lateral plate mesoderm (LM) lineages as described previously in^45,46,51^ and were further matured in SMC media with or without 0.2 μg/mL doxycycline (Dox) for a minimum of 5 days before phenotyping and molecular analyses.

### 2.2 Bulk mRNA-sequencing

Vec or RBPMS-hESC-VSMCs (both treated with Dox and untreated) were trypsinized, FACS sorted for GFP intensity into ‘low’, ‘medium’, and ‘high’ gates and used for RNA (Direct-zol, Zymo Research) and protein (Laemmli) isolation. In general, VSMCs obtained from multiple neural crest differentiations and/or independent hESC clones were considered as biological replicates. For bulk mRNA-sequencing, two rTTA-Vec-hESC clones with one differentiated into two NC lines (total 3×) and two rTTA-RBPMS-hESC clones differentiated into two separate NC lines each (total 4×) were used. For the RBFOX2 knockdown samples, three rTTA-RBPMS-hESC clones were used, and only the Dox-treated samples (control or anti-RBFOX2 siRNA) were sorted and sequenced. Analysis was conducted with the following pipelines—edgeR v3.28.10 for differential expression^52^ rMATS v4.0.2^53^ for splicing, and MATT v1.2.0^54^ for motif searches.

### 2.3 Cell motility

Live cell imaging of hESC-VSMCs was performed with the incucyte system every 2 h over the course of at least 8 h. Three and two clonal lines were imaged for the RBPMS-hESC-VSMCs and Vec-hESC-VSMCs, respectively. Cell tracking analyses (blinded) were performed using the imageJ manual cell tracking plugin (http://rsb.info.nih.gov/ij/plugins/track/track.html) with the ibidi cell tracker (Chemotaxis and Migration tool Ver 2.0).

### 2.4 Proliferation assays

Dox-treated RBPMS-hESC-VSMCs were either treated with 50 ng/mL of PDGF-BB or left unstimulated over the course of 72 h up to 96 h. Cells were then dosed with 10 uM EDU overnight, trypsinized, fixed with 4% PFA and analysed for EDU incorporation (Click-iT plus EDU kit—Thermo Fisher Scientific—C10634) and DNA content (Fx-Violet Thermo Fisher F10347 or DAPI) on a BD-Fortessa flow cytometer with area parameters enabled. Analysis was performed with Flowjo V10.7.1. A minimum of 1000 events was collected in each sample.

### 2.5 Statistics

Statistics were performed, and most graphical data were generated with GraphPad Prism ver7 and above.

## 3. Results

### 3.1 hESC-VSMCs do not endogenously express detectable levels of RBPMS protein

**Figure 1:**
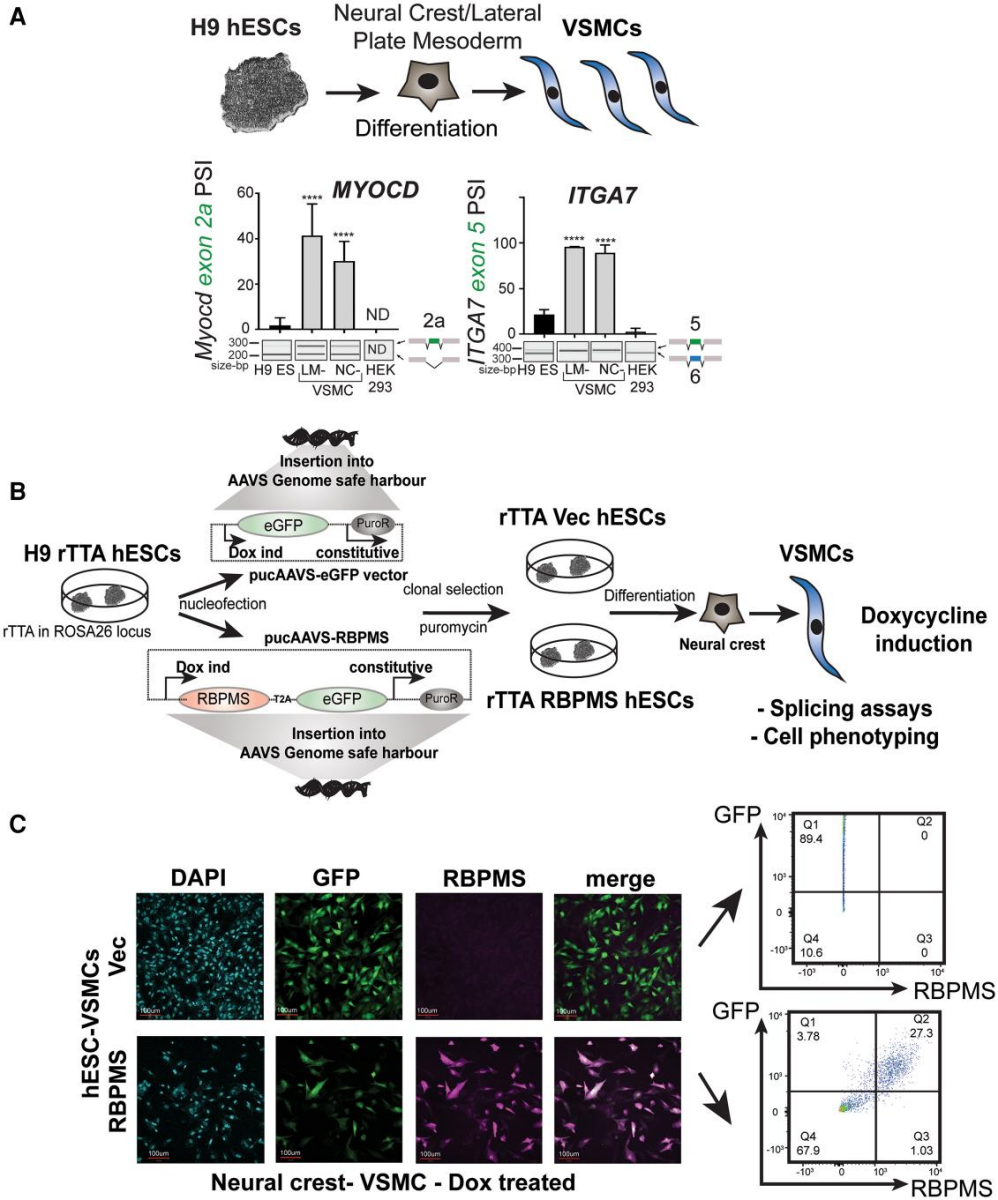
hESC-VSMC model to investigate RBPMS role in SM-AS. *(A*) H9-hESCs were differentiated into VSMCs via NC or LM lineages. RT–PCR of splicing targets *MYOCD* and *ITGA7* comparing NC and LM VSMCs (*N*≥3) with H9 (*N* = 2) and HEK-293 cells are shown. Graphs show PSI of the SM-specific exons (green - upper band in both targets shown) using data from the electrophoretic images below. ****P < 0.0001 with one-way ANOVA Dunnett’s multiple comparisons test. ND, not detectable. *(B*) H9-rTTA hESCs heterozygous for the rTTA protein in the ROSA26 locus were nucleofected with pUC-AAVS-eGFP vectors containing either GFP (rTTA-Vec-hESCs) or RBPMS-T2A-GFP (rTTA-RBPMS-ESCs) under the control of Dox-inducible promoters. Selected clones of both hESC lines were differentiated via the NC lineage into VSMCs, induced with 0.2 ug/mL Dox in 10% FBS containing SMC media for at least 5 days before splicing and phenotyping assays. *(C*) Representative immunofluorescence images showing rTTA Vec and rTTA-RBPMS-hESC-derived VSMCs via the NC lineage (*N* = 3). Mosaic GFP expression correlates with RBPMS protein levels in the RBPMS line. Scale bars: 100 um. Representative flow cytometry shows correlation of GFP and RBPMS staining in RBPMS-T2A-GFP-VSMCs (*N* = 3).

To determine the impact of RBPMS in human VSMCs, we used a hESC-derived model. hESCs were first differentiated into NC or LM intermediate lineages. Following this, the cells were subjected to PDGF-BB and TGFβ treatment in basal media for 12 days^45,46,51^ and then maintained in 10% FBS VSMC media to mature hESC-VSMCs. Compared to ESCs and HEK293T cells, hESC-VSMCs from both lineages not only display increased levels of several VSMC markers^45,46,51^ but also increased SM-AS patterns. Some genes, such as *ITGA7*,^11^ showed a complete switch to the SM-AS isoform (*Figure 1A*) similar to the pattern seen in differentiated or mature tissue VSMCs (see Supplementary material online, Figure  *S1A*). For others, such as *MYOCD*,^12^ the switch in splicing is only partial, with lower levels of exon inclusion (*Figure 1A*) than are seen in adult arterial tissue where exon inclusion was near 100% (see Supplementary material online, Figure  *S1B*). However, when we examined the hESC-VSMCs for RBPMS expression, we could not detect endogenous protein although baseline level of *RBPMS* transcripts was detectable by quantitative RT–PCR (see Supplementary material online, Figure  *S2A*). Rather, the levels of RBPMS generally decreased during differentiation from ESCs (H9 ESCs) to hESC-VSMCs via both the NC and LM lineages with the intermediate stages showing variable levels of the protein (see Supplementary material online, Figure  *S2B*). We hypothesized that *RBPMS* expression might be associated with mature adult VSMCs, whereas hESC-VSMCs are more embryonic-like. Consistent with this, human heart single-cell RNA-Seq data^7^ show that *RBPMS* is co-expressed with advanced marker *MYH11* in VSMCs and is in the top 5% of all significant positive correlations with MYH11 (rank 206 of 5320 genes, *P* < 0.05, Spearman’s Rho = 0.24; Supplementary material online, Figure  *S2C*–*E*). Similar to transcriptional regulator *MYOCD, RBPMS* expression correlates positively with VSMC markers but negatively with endothelial, cardiomyocyte, and epicardial markers (see Supplementary material online, Figure  *S2E*). It is therefore likely that RBPMS is preferentially expressed in mature adult VSMCs, but at much lower levels in immature foetal-like hESC-VSMCs.

### 3.2 Dox-inducible over-expression in hESC-VSMCs reveals RBPMS as a major driver of modulations in the transcriptome

We hypothesized that the immature AS patterns in hESC-VSMCs might result from the lack of RBPMS expression. To address this, we generated an inducible over-expression platform. We inserted constructs bearing GFP alone (Vector/Vec lines) or RBPMS (isoform A) linked to GFP via a T2A sequence (RBPMS lines) under the control of Dox-inducible promoters into the pUC-AAVS genomic safe harbour of the H9-rTTA ESC line.^50^ This hESC line is heterozygous at the ROSA26 locus for the reverse transactivator gene (rTTA) and constitutively expresses the rTTA protein. Puromycin-resistant Vec and RBPMS-hESC clones were expanded and differentiated via the LM or the NC intermediates into hESC-VSMCs. Dox (0.2 ug/mL) was added to the media to induce RBPMS expression, and cells were treated for at least 5 days before expansion and use for molecular and phenotypic analyses (*Figure 1B*).

**Figure 2:**
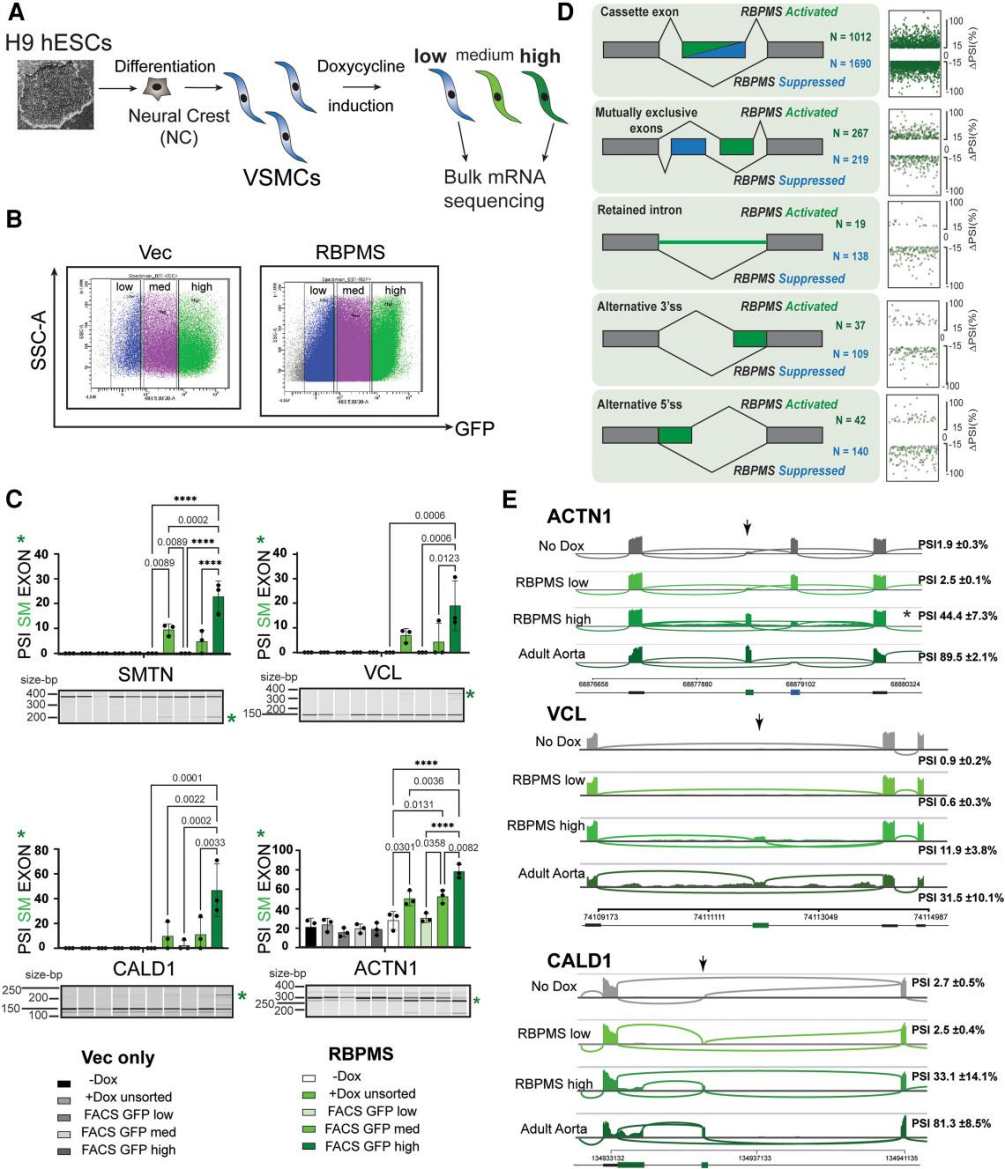
FACS sorted NC–derived VSMC fractions for GFP intensity show differential splicing patterns in a RBPMS dose-responsive manner. (*A, B*) Dox-treated, NC-derived Vec, and RBPMS-hESC-VSMCs were sorted for GFP intensity using flow cytometry. RNA was isolated and bulk mRNA-sequencing performed on GFP low and high fractions. *N* = 4 for RBPMS-hESC-VSMC and *N* = 3 for Vec-hESC-VSMC lines. *(C*) RT–PCR of selected SM-AS targets from untreated and Dox-treated unsorted, low, medium, and high GFP fractions of Vec and RBPMS-hESC-VSMCs (three independent NC differentiations of one Vec and one RBPMS clonal line). PSI of the SM exon is graphed with all targets tested showing RBPMS dose-responsive induction. Asterisk highlights the SM exon band in the electrophoresis image. Note: in SMTN, the SM isoform is an exon skipping event, whereas VCL, CALD1, and ACTN1 involve inclusion of an SM exon. Ordinary one-way ANOVA with Sidak correction for multiple comparisons was performed across samples within each cell type. ****P < 0.0001 with exact values indicated for ***P < 0.001, **P < 0.01, and *P < 0.05. Only statistically significant comparisons are indicated. *(D*) rMATS splicing analysis of RBPMS-high and RBPMS-low hESC-VSMCs reveals a network of splicing changes induced by RBPMS over-expression. Splicing patterns altered upon RBPMS over-expression cover >3000 high confidence (FDR < 0.05, |ΔPSI| ≥ 15%) events across five major alternative splicing categories. Schematics describe the type of splicing seen in each category whether RBPMS-activated (increased PSI with RBPMS i.e. positive ΔPSI) or RBPMS-repressed (decreased PSI with RBPMS i.e. negative ΔPSI). *(E*) Sashimi plots of RBPMS-regulated exons and the flanking constitutive regions in *ACTN1*, *VCL*, and *CALD1*. SM exons indicated by arrow above. No Dox (untreated), RBPMS-low, RBPMS-high, and adult human aorta^55^ tracks are shown. SM exon isoforms are increased in RBPMS-high compared to control conditions. SM exon PSI values are average ± standard deviation, *N* = 4 for No Dox, RBPMS low and high samples, *N* = 3 for adult aorta samples. For the mutually exclusive ACTN1 SM exon, asterisk indicates the discrepancy in PSI values predicted by rMATS and the exon expression visual in the tracks. The trend in increase in SM exon PSI, however, remains the same.

Dox-inducible over-expression was inherently and consistently mosaic. Cells within the same population expressed varying levels of GFP and corresponding levels of RBPMS (*Figure 1C*). This is likely due to partial and stochastic epigenetic silencing at the pUC-AAVS locus as sodium butyrate treatment (pan HDAC inhibition) allowed for GFP expression in all Dox-inducible cells (data not shown). We subsequently used the mosaicism to our advantage as an internal control allowing comparison of Dox-treated cells that express low and high levels of RBPMS under identical culture conditions. Dox-treated RBPMS-hESC-VSMCs were FACS sorted based on their GFP intensity, which correlated with RBPMS expression (*Figures 1C* and *2A* and *B*), and RNA was isolated from the various fractions. In addition, as a control, we used the vector lines that expressed GFP alone upon Dox induction and sorted these cells based on GFP intensity. RT–PCR for known targets of RBPMS-induced splicing^12^ showed that only high GFP/RBPMS expressing cells from the RBPMS-hESC-VSMCs displayed tissue-like contractile SM-AS. The corresponding low-GFP RBPMS-hESC-VSMC populations and both high- and low-GFP samples from the Vec-hESC-VSMCs displayed baseline AS. This was verified in VSMCs from both the NC (*Figure 2C*) and the LM (see Supplementary material online, Figure  *S3A*) lineages. hESC-VSMCs of the NC lineage were used for all subsequent experiments.

To obtain a global view of transcriptome changes induced by RBPMS, we performed bulk pre-mRNA-sequencing of untreated (No Dox) and Dox-treated (+Dox) low-GFP and high-GFP populations from Vec and RBPMS-hESC-VSMCs (*Figure 2A*). At the mRNA abundance level, we observed very few changes between No Dox and +Dox GFP-low RBPMS or Vec cells (<10 genes), indicating that Dox treatment was not having widespread unintended off-target effects. In contrast, RBPMS/GFP high hESC-VSMCs when compared with RBPMS/GFP-low VSMCs or untreated cells showed differential expression of 63 and 95 genes, respectively (edgeR log2FC ≥ 1.25 or < −1.25, *P*_Adj_ < 0.05—bulkAnalysR shinyApp^56^). The Vec-hESC-VSMCs showed minimal changes between No Dox and +Dox GFP high and low. We therefore focused our analyses on the tightly controlled comparison between GFP-high and GFP-low RBPMS cells.

### 3.3 RBPMS drives tissue-like contractile SM-AS in hESC-VSMCs

To investigate the global consequences of RBPMS expression on AS, we used rMATS,^53^ treating as significant only those AS events with cut-offs of FDR < 0.05 and Percent Spliced In difference (ΔPSI) > 15%. A total of 3673 AS events were differentially regulated between RBPMS-high and RBPMS-low cells, encompassing the five AS categories identified by rMATS (*Figure 2D*; Supplementary material online, table  *S1*). Known SM-AS events regulated by RBPMS included ACTN1, VCL, CALD1 (*Figure 2E*), SMTN, and TPM1 (see Supplementary material online, Figure  *S3B*); in each case, RBPMS promoted splicing patterns akin to adult human aorta contractile tissue VSMCs (data mined from GSE147028.^56^). The 15% ΔPSI threshold was used to reflect the probable low functional impact of small AS changes (e.g. from 40 to 55% PSI). However, some key contractile VSMC markers such as meta-VCL^14,15^ did not cross the ΔPSI threshold but are strongly induced in RBPMS-hESC-VSMCs; sashimi plots revealed clear changes in splicing of VCL, from an average of ∼0.6% to 11.9% PSI (FDR = 0), a 20-fold induction of the SM meta-VCL isoform (*Figure 2E*). Moreover, meta-VCL induction could be observed by immunoblotting (see Supplementary material online, Figure  *S3C*) and RT–PCR (*Figure 2C*), and even in adult aorta, the exon is only included to ∼30% (*Figure 2E*). Our data therefore demonstrate that RBPMS expression is sufficient to induce multiple AS changes, many of which attain patterns characteristic of adult aorta VSMCs.

**Figure 3:**
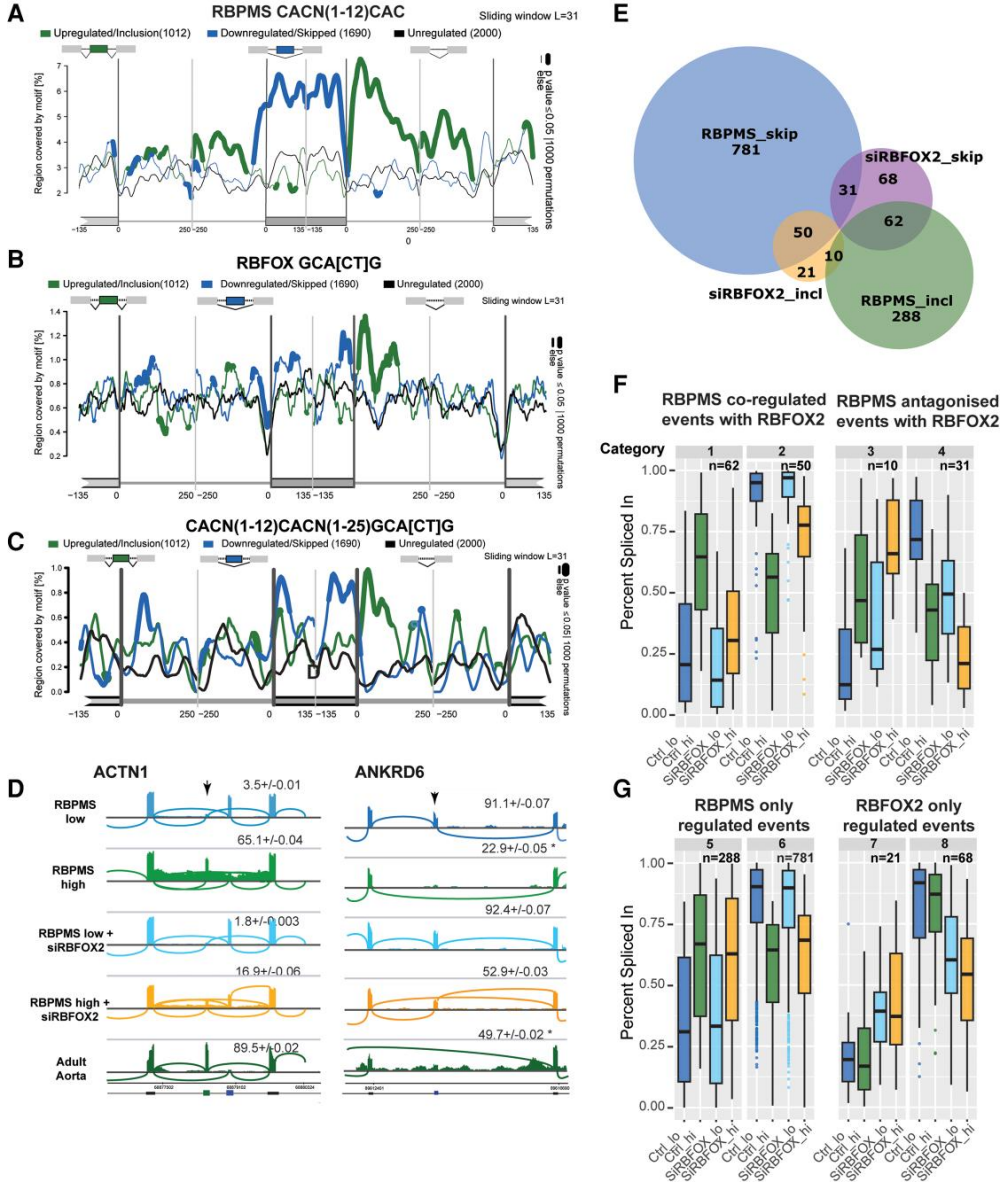
RBPMS-regulated exons show enrichment of CAC clusters indicating position-dependent splicing activity. *(A*) RBPMS-binding motifs (CACN_1–12_CAC) are significantly enriched in the downstream intron of RBPMS-activated exons and on RBPMS-repressed exons. RNA maps motif enrichment search with a sliding window length of 31 nucleotides using MATT. The test cassette exons were activated (*n* = 1012, upregulated/inclusion) or repressed (*n* = 1690, downregulated/skipped) by RBPMS (|ΔPSI| > 15%, FDR < 0.05). The reference set consisted of 2000 randomly selected non-regulated events (FDR > 0.1, |ΔPSI| < 10%). Statistically significant (permutation test 1000 iterations—*P* ≤ 0.05) occurrences on regulated exons (135 bp from the upstream and downstream ends, 250 bp of the flanking introns, and 135 bp the flanking constitutive exons) are marked by the thick lines with y-axis showing the % region covered by the defined motif. *(B*) RNA maps of RBFOX motif (GCAC/TG) enrichment around RBPMS-regulated and control exons. *(C*) RNA maps showing co-occurring RBPMS and RBFOX-binding motifs on RBPMS-regulated exons and their flanking introns. *(D*) Bulk mRNA-sequencing of siRBFOX2 or control siRNA-treated hESC-VSMCs (*N* = 3 independent RBPMS-hESC clones differentiated via NC lineage) was performed after sorting the Dox-treated cells for low or high RBPMS expression. Sashimi tracks of ACTN1 and ANKRD6 representing events where RBPMS directs splicing patterns towards the adult aorta status that are largely reversed by RBFOX2 depletion—i.e. RBPMS and RBFOX2 co-regulated events. ANKRD6* indicates a location of multiple alternative splicing events that skew the PSI (+/− SD) estimation by rMATS. However, it is clear that the non-SM exon (arrow) is skipped in adult tissue and in RBPMS over-expression. *(E*) rMATS analysis was performed comparing siRBFOX2 treated samples with cognate controls and events classified by regulation by RBPMS and/or RBFOX2. Venn diagram summarizing the overlap and *(F*, *G)*, box plots showing patterns of RBPMS and RBFOX2-regulated events. Events classified as regulated by RBPMS (Categories 5 and 6) or RBFOX2 (Categories 7 and 8) or both—co-ordinately (Categories 1 and 2) or antagonistically (Categories 3 and 4)—included or skipped showed ΔPSI ≥ 15% and FDR < 0.05. Average PSIs across three biological replicates are plotted.

To establish whether RBPMS acts as a direct splicing regulator, we searched within differentially spliced cassette exon events for enrichment of putative RBPMS motifs—pairs of CAC triplets separated by 1–12 nucleotides reflecting RBPMS-binding RNA as a dimer.^57^ We found significant enrichment of the RBPMS motifs with RBPMS-regulated exons, consistent with direct binding by RBPMS to regulate the splicing of its target exons (*Figure 3A*). Moreover, RBPMS motifs were highly enriched in the intron immediately downstream of exons that were included upon RBPMS over-expression and somewhat less so in the upstream intron. In contrast, exons that were skipped upon RBPMS over-expression showed the presence of RBPMS motifs primarily on the exon itself and in the immediate upstream intron overlapping the 3′ splice site (*Figure 3A*). This pattern is consistent with the position-dependent regulatory rules of other well-known splicing factors.^58^ To corroborate, we took a more agnostic approach by performing k-mer (8-mer) enrichment analysis on differentially spliced targets followed by alignment and hierarchical clustering of significantly enriched (*P* < 0.01) 8-mers. Consensus 5-mer motifs within the clusters also emerged as CAC-rich (see Supplementary material online, Figure  *S4A*) particularly in the downstream introns of RBPMS-included exons (see Supplementary material online, table  *S2*). Overall, the enrichment of known RBPMS motifs around its target exons is strongly consistent with the direct binding and regulation of these exons by RBPMS.

Splicing regulatory RBPs rarely function in isolation and more commonly act cooperatively or antagonistically with other RBPs. One notable 5-mer sequence that emerged from k-mer enrichment analysis was GCATG, which was significantly enriched downstream of RBPMS activated exons (enrichment score = 2.02, *P* < 0.001). This is a well-known splicing regulatory motif that specifically binds members of the RBFOX family of splicing regulators,^59^ which have similar position-dependent splicing activity as RBPMS. Indeed, ‘splicing maps’ revealed a significant enrichment of RBFOX motifs downstream of RBPMS-activated exons and immediately upstream of the 5′ splice site of RBPMS-repressed exons (*Figure 3B*), similar to the distribution of RBPMS binding CAC motifs (*Figure 3A*). Indeed, enrichment of linked RBPMS and RBFOX motifs (within 25 nt of each other) showed that the two motifs are enriched together on the same transcripts (*Figure 3C*). The specificity of association between the two motifs was demonstrated by the minimal enrichment when a single nucleotide change was made to the RBFOX motif (see Supplementary material online, Figure  *S4B*). RBFOX2 is the only family member that is highly expressed in VSMCs (see Supplementary material online, Figure  *S5A*). hESC-VSMCs express RBFOX2 protein, and RBFOX2 mRNA levels almost equal to adult tissue levels, that were unaffected by RBPMS induction (see Supplementary material online, Figure  *S5B*–*E*). The co-occurrence of their motifs therefore suggests that RBFOX2 and RBPMS functionally cooperate in VSMC splicing regulation. To test this possibility, we knocked down RBFOX2 in RBPMS-hESC-VSMCs with and without RBPMS over-expression and analysed selected AS events by RT–PCR (see Supplementary material online, Figure  *S5B*, *C*). In both ACTN1 and SMTN, RBFOX2 depletion reversed the effects of RBPMS over-expression resulting in loss of contractile splicing patterns (see Supplementary material online, Figure  *S5B*). To investigate the global relationship between the two regulators, we performed bulk mRNA-sequencing of RBPMS-hESC-VSMCs with RBFOX2 knockdown (see Supplementary material online, Figure  *S5D*, *E*) with or without RBPMS over-expression and analysed splicing changes with rMATS. The effect of RBPMS over-expression on splicing in control siRNA-treated hESC-VSMCs was consistent with observations from our first RNA-Seq data set, with highly correlated RBPMS splicing activity (see Supplementary material online, Figure  *S5F*). *Figure 3D* shows sashimi plots of example events in ACTN1 and ANKRD6, where RBPMS-driven adult tissue VSMC splicing is largely lost upon RBFOX2 depletion. RBPMS over-expression regulated ∼5 times as many AS events as RBFOX2 knockdown 1222 vs. 242 events (*Figure 3E–G*). However, ∼63% of the RBFOX2-regulated cassette exon events were also regulated by RBPMS, and in ∼73% of these events, RBPMS and RBFOX2 acted cooperatively rather than antagonistically. Notably, the co-regulated set of exons largely contributed to the observed enrichment of GCAUG with RBPMS-regulated exons (data not shown). The co-enrichment of RBFOX motifs with RBPMS regulated exons therefore reflecting co-regulation of a significant subset of exons by both proteins.

### 3.4 RBPMS-directed SM-AS affects functionally critical gene groups for adult aortic VSMCs

To investigate the potential biological consequences of RBPMS-regulated AS, we initially performed gene ontology (GO) analyses of affected genes, using a stringent ΔPSI cut-off of 30% (rather than 15%) to obtain a network of AS events that are likely to have functional impact. Enriched terms in RBPMS splicing targets included actin binding, focal adhesions, and contractile actin filaments (see Supplementary material online, Figure  *S6A*, *B*). In contrast, GO analyses of the differentially expressed genes induced by RBPMS over-expression did not feature these or any other significant or specific biologically relevant terms (data not shown). These results suggest that RBPMS is capable of remodelling the actin cytoskeleton, potentially to suit the contractile state of VSMCs via changes in splicing patterns rather than by modulating the mRNA abundance of the various component genes.

**Figure 4:**
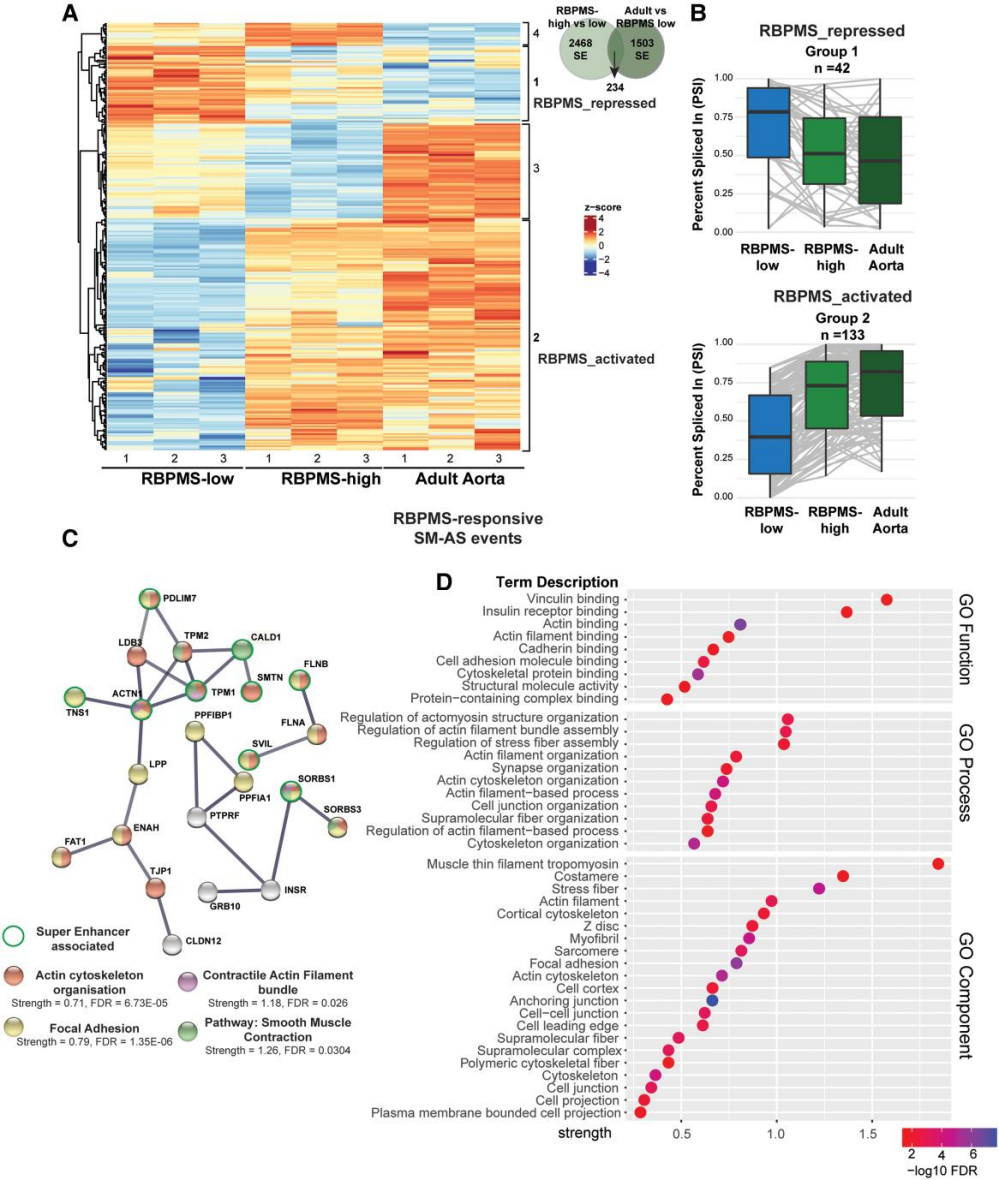
RBPMS promotes tissue VSMC AS patterns. *(A*) Heatmap of 234 cassette exon events significantly regulated between human aortic tissue and hESC-VSMCs and also regulated by RBPMS (Venn diagram). Clusters 1 and 2 are events where RBPMS regulation matches aortic tissue splicing. *(B*) Box plots summarizing the relative splicing pattern of the events included in clusters 1 and 2 of the heatmap in A. *(C*) Protein–protein interaction network with StringDB of the RBPMS-regulated aorta SM-AS events from B combining genes from clusters 1 and 2. High confidence nodes of interaction scores 0.7 or more (whole genome background) are shown covering key factors involved in the contractile, focal adhesion, and actin cytoskeletal machinery. Key shows colour code for the nodes and the enrichment strength and FDR values (Benjamini–Hochberg). Nodes outlined with the bold circles indicate genes associated with super-enhancers in the human aortic tissue (https://asntech.org/dbsuper/). *(D*) Dot plots showing top enhanced enriched terms of clusters 1 and 2 in the molecular function, cellular component, and biological process categories, respectively. The terms enriched are represented with −log10 FDR and filtered from StringDB analyses for term strength (log 10 observed/expected) of 0.1 or more and FDR ≤ 0.001.

To put the RBPMS-regulated splicing network in the context of the AS network of adult human aortic VSMCs, we mined bulk mRNA-sequencing data sets of normal adult human aorta.^56^ We performed rMATS analysis comparing RBPMS-low hESC-VSMCs with the adult aorta and obtained 1737 significantly alternatively spliced skipped exon (SE) events with at least 15% ΔPSI. This network showed enrichment of terms including actin cytoskeleton and focal adhesion, similar to the RBPMS regulated AS network (see Supplementary material online, Figure  *S7A*). To delineate the influence of RBPMS in this network, we isolated the commonly differentially spliced events shared by the RBPMS and the adult aorta splicing networks. This yielded 234 SE events that were significantly differentially spliced in both data sets (*Figure 4A*). Strikingly, clustering of these events by Percent Spliced In (PSI) values showed that RBPMS induced the mature contractile splicing pattern in 75% (175 of 234) of the events (*Figure 4B*, Supplementary material online, table  *S1*). Of these events, 133 were activated by RBPMS (Group 2) and 42 were repressed (Group 1). This represents ∼10% of the adult aorta splicing network and is statistically significant (hypergeometric test, *P* = 2.0e−35—pHyperR). RBPMS over-expression in hESC-VSMCs brought the PSI of regulated AS events close to the value found in tissue VSMCs (*Figure 4B*). Significantly, a subset of 28 events could be detected where RBFOX2 and RBPMS co-ordinately promote adult aorta splicing patterns (see Supplementary material online, Figure  *S7B*). The two smaller groups of events with discordance between RBPMS and tissue vs. hESC-VSMC regulation might represent events that are not authentic contractile VSMC markers but instead arise from the comparison of tissue with cultured cells and possibly the over-expression levels of RBPMS (see Supplementary material online, Figure  *S7C*). These genes were not associated with any specific GO terms and a minimal protein–protein interaction network (see Supplementary material online, table  *S3*). In contrast, GO and protein network analyses of the 175 events where RBPMS promoted tissue like VSMC patterns showed enrichment of terms pertaining to actin cytoskeleton organization and focal adhesions—all connected to smooth muscle contractility and similar to the GO terms enriched in the SM-AS network (*Figure 4C, D*). Interestingly, of the 144 genes containing the 175 RBPMS-regulated adult aorta SM-AS events, 21 (*Figure 4C*, Supplementary material online, table  *S3*) were associated with super-enhancer regions defined in adult human aortic tissue (703 total in aorta -dbSUPER database),^60–62^ a significant overlap (*P* = 9.7e−09, pHyperR). Given that super-enhancers are associated with genes that are critical for cell type identity,^63^ this observation argues that RBPMS regulates AS events in genes that are crucial for mature VSMCs.

### 3.5 RBPMS over-expression alters proliferation and motility in hESC-VSMCs

**Figure 5:**
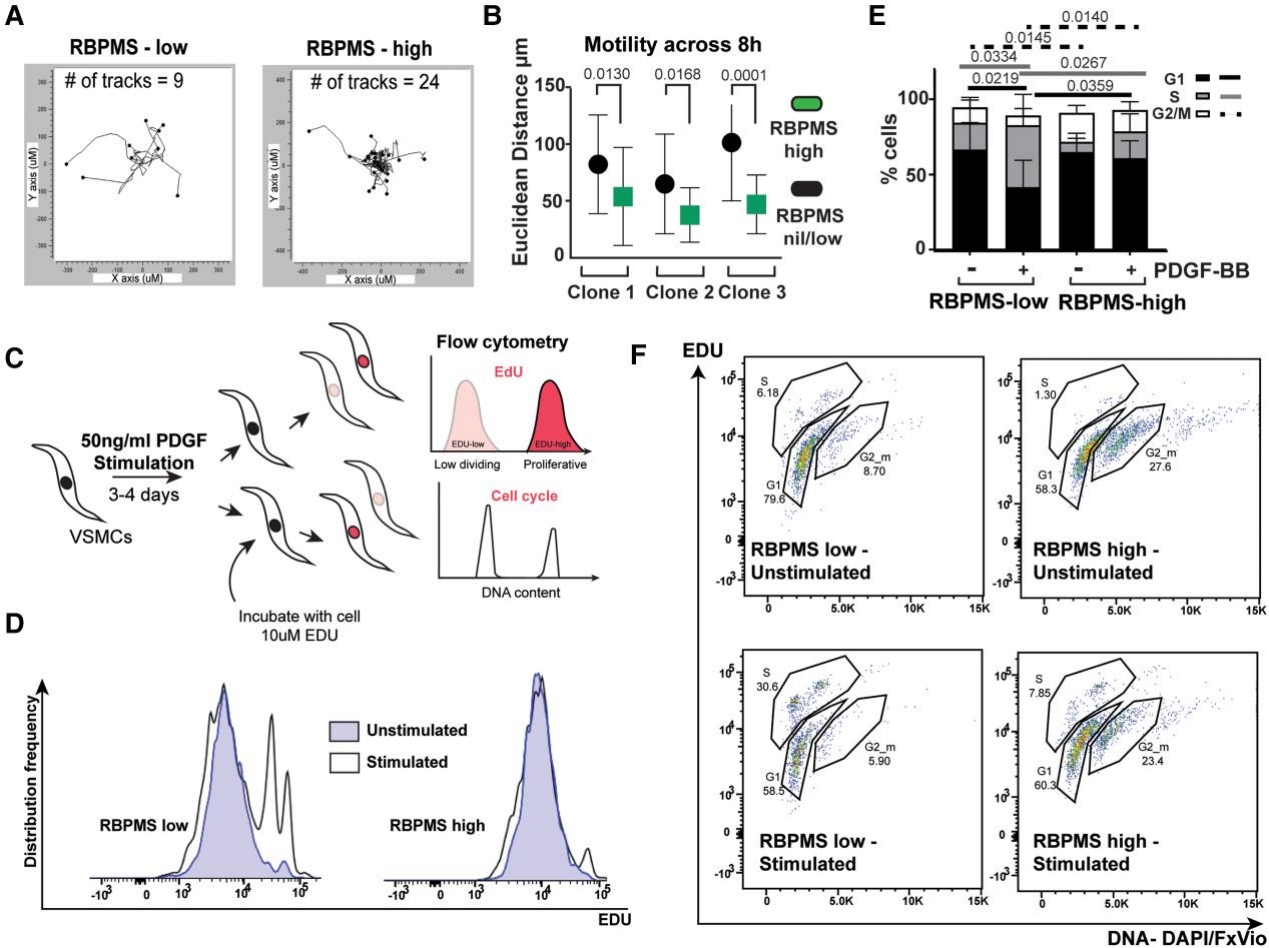
RBPMS over-expression alters hESC-VSMC phenotypic behaviour. *(A*) Motility of Dox-treated RBPMS-hESC-VSMCs was examined with live cell imaging over the course of 8 h. Representative cell trajectory plots (ImageJ cell-tracker and Ibidi software) showing 9 RBPMS-low 24 RBPMS-high cells displaying differential motion. *(B*) Euclidean distance traversed by 40 GFP positive and 40 GFP negative hESC-VSMCs (across duplicate wells) derived from three independent RBPMS-hESC clones. RBPMS-high cells are less motile than RBPMS-low cells (two-way ANOVA multiple comparison without correction independent Fisher’s LSD tests, *P* values indicated). *(C*) Experimental scheme for hESC-VSMC proliferation assay. *(D*) Representative distribution frequency histograms (FlowJo population comparison) showing EDU incorporation in PDGF-BB stimulated and unstimulated hESC-VSMCs gated for low (RBPMS-low) and high GFP (RBPMS-high). PDGF-BB stimulates EDU labelling in RBPMS-low cells, but this is substantially reduced in RBPMS-high cells. *(E*) Distribution of RBPMS-high and RBPMS-low cells (% parent population) with or with PDGF-BB stimulation across the cell cycle. RBPMS-high cells do not enter S phase as much as the RBPMS-low cells and show higher baseline G2/M populations. Statistical significance of the most meaningful comparisons across the different groups is indicated with dark lines (G1 phase differences), light lines (S phase differences), and dashed lines (G2/M differences). Fisher’s LSD test without correction for multiple comparisons in two-way ANOVA—*P* values indicated. Data summarize the analyses from seven independent EDU incorporation assays using hESC-VSMCs derived from two separate RBPMS-hESC clones. Biological replicates derived from at least two independent NC differentiations per clone. *(F*) Gating strategy of a representative hESC-VSMC-PDGF–induced proliferation assay. These data correspond to the populations compared in the histogram in (*D*). Double staining for incorporated EDU and DNA content which allows gating for the populations in different cell cycle stages.

Given that focal adhesions and cytoskeleton related terms featured prominently in GO analyses, we sought to examine phenotypic features of RBPMS over-expressing hESC-VSMCs that might be affected by alternative splicing in these complexes. When we compared the motility of RBPMS-high vs. RBPMS-low hESC-VSMCs in the same Dox-treated population, we observed that RBPMS-expressing cells were significantly less motile (lower mean Euclidean distance) than their low RBPMS counterparts when we followed their motion over the course of 8 h using live cell imaging (*Figure 5A, B*). GFP-high and GFP-low Vec-hESC-VSMCs did not differ significantly in their motility (see Supplementary material online, Figure S*S8*). These results suggest that RBPMS-induced splicing changes are capable of driving phenotypic alterations in hESC-VSMCs similar to mature, healthy arterial VSMCs which are also non-motile.^64^ However, RBPMS over-expression did not cause any perceivable or overt cytoskeletal rearrangements (data not shown), in contrast to the effects of its depletion in adult rat PAC1 VSMCs.^12^

In addition to changes in cytoskeleton-related genes, RBPMS also influenced the splicing of a small cluster of cell cycle–associated genes (see Supplementary material online, table  *S1*). We queried the proliferative capacity of RBPMS-high cells in comparison with RBPMS-low cells using EDU labelling (*Figure 5C–F*). We first induced proliferation in the hESC-VSMCs by treating them with the mitogen PDGF-BB for at least 72 h. Following this, we labelled the cells with EDU and performed flow cytometry to examine DNA content in addition to EDU incorporation (*Figure 5C*). We observed that RBPMS-high cells in general displayed lower EDU incorporation when stimulated with PDGF-BB when compared to the RBPMS-low cells (*Figure 5D*). In addition, a smaller percentage of RBPMS-high cells entered S-phase after PDGF-BB treatment (*Figure 5E, F*). There was also a higher proportion of RBPMS-high cells in G2/M phase both at baseline and PDGF-stimulated conditions. This result was reproducible in two independent RBPMS-clonal lines assayed across seven independent differentiations. This indicates that RBPMS over-expression alters the proliferative properties of hESC-VSMCs pushing them towards a more mature, less proliferative phenotype, again similar to the properties of healthy arterial VSMCs.

## 4. Discussion and conclusions

RBPMS and its paralog RBPMS2 have recently come to prominence as key regulators of cardiac development.^38–40,44^ We previously showed that RBPMS is also a key driver of the differentiated VSMC phenotype in rat PAC1 cultured cells,^12^ while single-cell studies have indicated that RBPMS is itself a marker of murine contractile VSMCs.^2^ Here, we show that RBPMS expression induces a mature contractile adult AS program in hESC-VSMCs, accompanied by decreased motility and proliferation, indicating that the role of RBPMS in VSMCs is conserved in humans.

hESC-VSMCs are inherently immature and foetal-like in their molecular profiles. Their differentiation from embryonic stem cells to VSMCs stimulates increased levels of some identity markers (e.g. *CNN1, TAGLN,* and *α-SMA*), but more advanced markers of VSMC maturity (e.g. *MYH11*) usually remain below detection levels.^46^ Similarly, while a small number of AS events (e.g. ITGA7, *Figure 1*) do acquire their mature pattern in hESC-VSMCs, the majority had basal SM-AS levels. Our original intention was to knock down RBPMS in hESC-VSMCs; however, its expression was also basal. Notably, our original identification of RBPMS as a potential master regulator of SM-AS was based upon the mapping of transcriptional super-enhancers to *RBPMS* in adult human aorta.^12^ We therefore turned to inducible over-expression and compared the resultant RBPMS-driven splicing program to an ‘adult VSMC’ programme defined by comparison of hESC-VSMCs with publicly available RNA-Seq data for adult human aorta. There are important caveats with both individual comparisons, but they combine to identify a functionally important core set of splicing events that provide a complementary, quantitatively distinct, set of phenotypic markers. Unlike transcriptional markers, whose expression can vary by several orders of magnitude with no clearly defined upper limit, AS events vary between set boundaries (0–100 PSI). Events such as meta-VCL, which only achieve 30% PSI in adult VSMCs, therefore provide quantitatively defined markers for mature VSMC phenotype.

The main limitations of RBPMS over-expression are that very high levels of a single RBPMS isoform are expressed. RNA-Seq data indicated that ‘RBPMS-high’ VSMCs had approximately five times the level of RBPMS levels found in adult aorta (see Supplementary material online, *Figure S3D*). Moreover, the overexpressed canonical RBPMS-A isoform is more active than the RBPMS-B protein isoform that usually accounts for a significant proportion of cellular RBPMS.^12^ It is therefore likely that some of the AS changes induced by RBPMS over-expression are not physiological targets. The main limitation of the defined adult VSMC program is the comparison of aortic tissue with cultured hESC-VSMCs. To properly map the adult SM-AS network, we would need to compare its splicing profile with a cognate phenotypically switched network *in vivo*, for example, during injury response.^5,65^ This is achievable in rodent systems but very challenging with human VSMCs. The AS network derived by comparison of aortic tissue with hESC-VSMCs likely includes not only AS events associated with VSMC phenotypic plasticity, including some that are driven by the higher levels of RBPMS in adult aorta compared to RBPMS-low hESC-VSMCs, but also others arising from artefactual differences arising from culture conditions, matrix stiffness, and other non-physiological factors. However, by focusing on splicing events that are regulated congruently by RBPMS over-expression and in adult aorta, we were able to circumvent the limitations of the individual comparisons and identified a core set of AS events that regulate a tight network of proteins involved in cell motility, contraction, and cell adhesion (*Figure 4*). This included dramatic increases in the expression of heavy-CALD1 and meta-VCL (*Figure 2*; Supplementary material online, Figure  *S3*), acto-myosin cross-linkers associated with mature, arterial tissue VSMCs as markers of contractility.^13–15^ The fact that this subset of the RBPMS-driven hESC-VSMC splicing was enriched for aortic tissue super-enhancer–associated genes (*Figure 4C*) increases confidence that it represents a key component of the human mature SM-AS programme. Like other RBPs, RBPMS has regulatory roles at multiple stages of gene expression from transcription^66,67^ to translation in ES cells,^68^ so it is possible that it has functional consequences in VSMCs beyond its splicing network. However, we observed minimal effects of RBPMS on mRNA abundance (shinyApps), with some of the observed changes due to AS events linked to nonsense-mediated mRNA decay (e.g. *PTBP2*,^69^  Supplementary material online, table  *S1*). Nevertheless, a critical link between RBPMS-regulated SM-AS and the mature VSMC transcriptional network is the transcriptional coactivator myocardin (MYOCD). MYOCD transcriptionally upregulates *RBPMS* in VSMCs leading to SM-AS induction upon over-expression.^70^ RBPMS, in turn, activates inclusion of the VSMC-specific exon 2a in *MYOCD*,^12^ which generates an N-terminally truncated isoform that is a stronger co-activator with SRF of CArG box genes in cell culture assays.^18^ It is therefore likely that a cross-regulatory nexus between transcriptional and splicing regulatory networks is an important component of the VSMC program. The degree and nature of such cross-talk remain to be explored more fully.

Although individual RBPs can act as master regulators that are responsible for driving cell-specific splicing programmes via changes in their own expression levels,^63^ such proteins usually work in cooperation with more widely expressed regulators. The significant co-enrichment of RBFOX-binding GCAUG motifs as well as RBPMS (CAC)_2_ motifs with RBPMS-regulated exons (*Figure 3*) strongly suggests that these two proteins act cooperatively. The GCAUG motif is highly specific for RBFOX1–3 family of RNA-binding splicing regulators.^26,71^ RBFOX1 and RBFOX2 are both active in skeletal and cardiac muscle during development, and their deregulation has been implicated in the aetiology of several cardiac pathologies.^26^ Knowledge of their splicing activity in VSMCs is limited to examples such as ACTN1^72^ and calcium channel CaV1.2, implicated in the pathology of hypertension.^73,74^ We focused on RBFOX2, which is the only family member expressed in hESC-VSMCs and human SMC tissues (GTEX; Supplementary material online, Figure  *S5A*). RBPMS physically interacts with RBFOX2 and co-regulates a number of target AS events in PAC1 cells.^75^ The RBFOX2 depletion RNA-Seq experiments in hESC-VSMCs confirmed that RBFOX2 is a necessary co-factor for a significant subset of SM-AS events (*Figure 3D–G*) that modulate the contractile, cytoskeletal, and focal adhesion machineries. Notably, while RBPMS levels are ∼5-fold higher in adult aorta compared to hESC-VSMCs (see Supplementary material online, Figure  *S3D*), levels of RBFOX2 are very similar (see Supplementary material online, Figure  *S5E*), supporting the concept that RBPMS is the critical cell-specific component that drives VSMC splicing. RBFOX2 itself is known to act via a Large Assembly of Splicing Regulators (LASR) complex,^76^ so it is possible that RBPMS co-opts the activity of a battery of splicing regulators via its intrinsically disordered C-terminal region^75^ to mediate VSMC-specific regulation.

We hypothesized that RBPMS-induced splicing changes in genes such as TPM1, PDLIM7, FLNB, ACTN1, TNS1, and SMTN might modulate the activity of the focal adhesion and actin-associated contractile and cytoskeletal machineries (*Figure 4C*), causing changes in motility associated with phenotypic modulation. Focal adhesion proteins mediate complex connections between the cytoskeleton and extra-cellular matrix (ECM) affecting the cell’s interaction with its environment and it is likely that alternative splice isoforms of these would mediate differential behaviours. For example, the SM-AS meta-VCL isoform has different F-actin bundling properties^77^ which bears implications for mechano-transduction, focal adhesion properties and cell motility.^78^ RBPMS over-expressing cells were indeed less motile (*Figure 5*) reflecting the low migratory properties of mature VSMCs. The contribution of individual alternatively spliced focal adhesion and cytoskeletal protein isoforms to this phenotype remains to be explored. In contrast to the effects of RBPMS depletion in partially differentiated PAC1 cells, where the alterations in AS affecting cytoskeletal protein isoforms correlated with observable changes in actin organization,^12^ we observed no changes in actin distribution upon RBPMS induction in hESC-VSMCs (data not shown). Although over-expression affected many of the same AS events (in the opposite manner to knockdown), it is possible that the splicing changes are necessary but insufficient for wholesale actin reorganization.

Mature VSMCs are quiescent and non-proliferative unless triggered by injury or insult to the vessel wall. In contrast, hESC-VSMCs are proliferative, particularly with mitogenic stimulation with PDGF-BB. RBPMS over-expression partly suppressed the proliferation of hESC-VSMCs with specific effects on S-phase entry and G2/M. This observation complements the increased proliferation of VSMCs during development in an *Rbpms* knockout mouse^40^ and the anti-proliferative action of RBPMS in breast cancer cells.^66^ RBPMS increased the inclusion of exon 30 of *FLNB* (see Supplementary material online, table  *S1*), whose skipping is a marker of EMT,^79^ suggesting that RBPMS promotes a more quiescent, non-mesenchymal state, whereas phenotypically switched VSMCs represent mesenchymal states.^1^

Rbpms homozygous knockout mice^40,80^ show pre-weaning/neo-natal lethality with 100% penetrance. The lethality was attributed at least in part to under-developed myocardium due to impaired cardiomyocyte proliferation at critical embryonic stages, associated with mis-splicing of PDLIM5. In contrast to the cardiomyocyte phenotype VSMC proliferation was enhanced in the developing vasculature coupled with a failure of ductus arteriosus closure after birth, i.e. patent ductus arteriosus.^40^ This is possibly due to the failure to transition to a contractile VSMC phenotype, which is necessary for ductus arteriosus closure.^40,81^ This supports the importance of RBPMS in the transition from a proliferative to a mature differentiated contractile state in developing and post-natal vasculature. To establish a direct relationship between RBPMS-regulated pathways and adult VSMC phenotypes, conditional knockout strategies will be necessary in combination with intricate single-cell sequencing studies.

**Translational perspective**

Since VSMC phenotype switching is a key factor in the pathology of diseases such as atherosclerosis, aneurysmal syndromes, hypertension, and even restenosis, it becomes critical to understand how these phenotypic states are achieved and maintained. Currently these states are primarily defined by the transcriptional profiles of these cells. This study shows that the RBPMS-driven mRNA splicing programme is an important post-transcriptional driver of mature VSMC phenotype.

## Supplementary Material

cvae198_Supplementary_Data

## Data Availability

The data presented in this study are available on the Gene Expression Omnibus under accessions GSE153614 (first experiment) and GSE242826 (second experiment). The datasets can be further explored using bulkAnalyseR apps^55^ on https://mohorianulab.org/shiny/sinha/RBPMS/GSE153614/ and https://mohorianulab.org/shiny/sinha/RBPMS/GSE242826/. Detailed procedures are in supplemental methods.
