# Supplementary material for: RNA binding protein with multiple splicing (RBPMS) promotes contractile phenotype splicing in human embryonic stem cell–derived vascular smooth muscle cells
Source: Cardiovasc Res. 2024 Sep 9;120(16):2104–16. doi: 10.1093/cvr/cvae198 (PMC11646123; doi:10.1093/cvr/cvae198)
Supplement: cvae198_Supplementary_Data [file cvae198_supplementary_data.zip › Supplemental Methods_V12.2_clean.docx]

**Supplemental Methods:**

**Doxycycline inducible system:** The H9-rTTA human embryonic stem cells (hESCs) as described in [1-3] were used to insert a cassette containing either GFP or RBPMS (isoform A [4]) cDNA linked to GFP via a T2A linker sequence under the control of Doxycycline-inducible promoters, into the pUC-AAVS genomic safe harbour locus. Briefly, hESCs were dissociated into single cells and electroporated with plasmids containing Zinc finger nucleases along with the pUC-AAVS constructs bearing the relevant cassettes. Positive clones were selected with 1ug/ml puromycin treatment and isolated and expanded as individual clones of either rTTA-Vec or rTTA-RBPMS hESCs. Clones were eventually expanded and maintained on Vitronectin coated plates and E8 media without puromycin (we did not observe any drift and clones were stable in Puromycin over multiple passages). Clones were validated for pUC-AAVS locus activity with 1ug/ml doxycycline treatment and GFP induction.

For differentiation to neural crest lineages, clones were “acclimatised” to 0.1% gelatin coated plates for 2 or 3 passages and then differentiated. Briefly, hESCs were seeded 24h prior to differentiation on vitronectin (LM only) or 0.1% Gelatin coated plates in “E8” media containing. Following this, the cells were treated in FSB media for 5 days to direct them toward NC lineage or with a combination of FlyB (36h) and FB (84h) for the LM lineage. For NC, cells were further passaged a minimum of 5 times and a maximum of 15 passages in FSB before use for VSMC differentiations. In the case of LM, once the intermediate was obtained, cells were directly used for VSMC production. LM or NC cells were treated for 12 days in PDGF-B and TGF-B media (PT) to generate VSMCs. hESC-VSMCs were then matured in SMC media containing 10% FBS with or without Doxycycline (0.2ug/ml) for a minimum of 5 days (to induce RBPMS and GFP expression).

Media recipes are all described in [5-8]. Briefly - Basal chemically defined media (basal media) - (Ham’s F12 + IMDM with concentrated lipids (Thermofisher 11905031), 15mg/ml Transferrin (R&D systems 3188-AT-001G), 0.1% PVA (Merck P8136), 7ug/ml insulin (Sigma Aldrich 11376497001). FSB – Basal media containing 12ng/ml FGF2 (R&D systems 233-FB) and 10nM SB431542 (Sigma Aldrich S4317), FlyB – Basal media containing 20ng/ml FGF2, 10uM LY294002 (Sigma Aldrich L9908) and 10ng/ml BMP4 (R&D systems 314-BP). FB – Basal media containing 20ng/ml FGF2 and 50ng/ml BMP4. PT – Basal media containing 10ng/ml PDGF-BB (Peprotech 100-14B-250) and 2ng/ml TGF-b (Peprotech 100-21-100).

**SiRNA knockdown:** RBPMS and Vec-hESC-VSMCs were differentiated from neural crest intermediates and treated with or without Doxycycline (0.2ug/ml) as above. Following this, cells were treated with either control (C2) or RBFOX2 siRNA (siRBFOX2) with Dharmafect 1 reagent (Dharmacon-Horizon T-2001-02) according to manufacturer’s protocols. Cells were transfected with 100pmol of Ctrl or anti-RBFOX2 siRNA per well of a 6-well plate for 72 hours and then treated a second time with the same dose for another 48h following which, they were harvested for RNA (Zymo – Directzol kit) and protein. C2 ctrl was used as in [4] and siRBFOX2 – Life technologies silencer predesigned siRNA- ID#136600 - GCCUUUUACUACCAUCCCAtt.

**Splicing PCR assays:** For RT-PCR splicing analyses, a minimum of 50ng of RNA was used to prepare cDNA (Roche Transcriptor RT 3531295001) and splice isoform specific PCRs (60deg annealing temperature, 35 cycles typically, MYOCD - 40 cycles) were performed (Sigma Aldrich Jumpstart High fidelity Taq D9307) using appropriate primers (see table below). For CALD1, a three-primer PCR approach was used to capture the alternative 5’ splice site event (For2) and a cassette exon event. PCR products were resolved using Qiaxcel capillary electrophoresis. Relative quantitation of splice isoforms was done calculating the relative band intensity percentage with Qiaxcel software.

| **Gene** | **Primer Sequence – 5’-3’** |
| --- | --- |
| MYOCD | For-GGCTAACCAAGGCATAATACC Rev-GAGATCATCGGCGAGTCG |
| ITGA7 | \| For- GATTGGTCGCTGCTTTGTGCTC \| \| --- \| \| Rev- TCTGCACGCACCAGACCTTTC \| |
| ACTN1 | For- CAGATCCTGACCCGGGATG Rev- CATGACTTGGTCTGCTGTATCTGT |
| CALD1 | \| For1- CAAAGCGAGGGAGCATTGGAG \| \| --- \| \| For2- CTTCAGACAGCTGTCCTAAAGAAAC \| \| Rev- TGTGGGTCATGAATTCTCCA \| |
| SMTN | \| For- GTGCACAACTTCTTCCCTGAG \| \| --- \| \| Rev- AGGTGGTTGTAGAGCGACTGC \| |
| VCL | \| For- TGAAGCTCGCAAATGGTCCAGCAAG \| \| --- \| \| Rev- ACCTCATCTGAGGCCTTGGCGATGT \| |
| RBPMS | \| For- AAAGCCGAGAAGGAGAACACC \| \| --- \| \| Rev- GGTCTGAAAAGCAGATAGAGCTCCC \| |
| GAPDH | \| For- AACAGCCTCAAGATCATCAGC \| \| --- \| \| Rev- GGATGATGTTCTGGAGAGCC \| |

**Antibodies and immunoblotting**: Immunoblotting was carried out using standard protocols. Protein samples were separated on 12-15% SDS-PAGE gels and transferred onto PVDF membranes and then probed for expression of specific proteins. Antibodies used were anti-RBPMS (Atlas antibodies HPA056999, 1:500 dilution for immunoblotting and 1:250 for flow cytometry and immunocytochemistry), anti-RBFOX2 (Atlas antibodies HPA006240 – 1:500 dilution), Vinculin (Sigma Aldrich v9264, 1:1000 dilution), GAPDH (Sigma Aldrich G9545, 1:5000 dilution).

**Flow cytometry and immunocytochemistry for RBPMS expression:** For RBPMS intra-cellular staining, cells fixed in 4% PFA were permeabilized using the BD Biosciences Cytofix/Cytoperm kit (554714) and stained with the RBPMS atlas antibody (same as for immunoblotting) overnight and then with Alexafluor 647 conjugated anti-rabbit secondary antibody (1:400). For immunocytochemistry, cells were fixed in 4% PFA, permeabilized with 0.1% Triton X-100 and stained with the RBPMS atlas antibody as above and then with Alexafluor 647 conjugated anti-rabbit secondary antibody (1:400) and imaged with a confocal microscope at 10x magnification.

**Library preparation:** of poly-A tailed RNA was performed with the NEB PolyA kit (E7490) and the NEB Ultra II directional library kit (E7760). 100ng of total RNA was used and initial quality control was done with Agilent RNA tapestation / RNA bioanalyzer reagents, and with the Qubit RNA HS Assay Kit (Q32855). Quality Control of the libraries was done with Qubit dsDNA HS Assay Kit (Q32854) and Agilent DNA 5000 tapestation reagents. Two randomised pools (of 11 and 10 samples) were sequenced on two lanes [9] of a Novaseq 6000 S1 flowcell, PE150 (Cancer Research UK, Cambridge).

**Bulk mRNA sequencing analysis pipelines:** For all analyses, quality checks were performed using FastQC (<https://www.bioinformatics.babraham.ac.uk/projects/fastqc>). The alignment to the reference genome (*H. sapiens* hg38, genome assembly GRCh38.p13) was done using STAR version 2.5.2a [10]. Pre- and post- alignment quality checks were summarised using MultiQC. Gene expression counts were generated using featureCounts version v1.6.0 (C). Additional quality checks include MA plots, PCA plots and heatmaps representing the Jaccard Similarity Index (JSI) [11].

**Bulk mRNA sequencing noise analysis pipelines:** The noise identification (on summarised counts and transcripts) and subsequent correction were performed using noisyR [12]. Pearson Correlation was used to determine the signal to noise transition; a correlation threshold of 0.25 between expression profiles across transcripts (transcript approach) and sorted gene expression vectors (count matrix approach), respectively was used for identifying the dataset specific signal to noise value. For the latter, the length of the vectors (sliding windows) was set to one tenth of the total number of transcripts. Both approaches yielded similar signal/noise thresholds (subsequent analyses were based on a noise threshold of 34). All transcripts with abundances less than the signal/noise threshold across all samples were excluded from subsequent analyses. The transcripts with at least one entry above the signal/noise threshold were kept for further analyses. All entries lower than the signal/noise threshold were increased to the threshold, to avoid false positive calls on the DE step.

The normalisation of expression levels was performed using quantile normalisation [13] (using the function normalize.quantiles() from the R package preprocessCore (https://github.com/bmbolstad/preprocessCore)). The differential expression (DE) analysis was performed using the standard functions from edgeR pipeline, version 3.28.0 [14]. The DE analysis was performed on the comparison of Test High against Test Low. The thresholds for DE were |log_2_(FC)| >0.5 and adjusted p-val < 0.05 (adjustment done using the Benjamini-Hochberg method). The differentially expressed genes were summarised in a volcano plot. Enrichment analysis was performed using g:profiler (R package gprofiler2, version 0.2.0) [15], against the standard GO terms, and the KEGG [16] and reactome [17] pathway databases. The observed set consisted of the DE genes, the background set comprised all expressed genes, using the denoised count matrix.

All steps of the analysis can be further explored using a bulkAnalyseR Shiny app [18], and also UCSC genome browser tracks created for this dataset.

**Splicing analysis:** Alternative splicing analysis in Control (Ctrl) and Test samples was performed comparing GFP high, GFP low or No-Doxycycline samples using rMATS [19] (version 4.0.2) with the BAM files filtered on noise thresholds as described above. The splicing profiles of adult human aorta tissue were obtained from GSE147026 - SRR11310798, SRR11310799, SRR11310800 [20]. The fastq files obtained from this study were processed and filtered using the same pipeline described above. Similarly noise filtered BAM files were used as input for rMATS. Raw rMATS outputs (on JC files) were then filtered for events that showed statistical significance using an FDR threshold of 5% and an inclusion level difference threshold of 15%. Further filtering on events for which there were at least 80 occurrences in each sample set (either inclusions or skipped junction reads) was applied, to exclude potentially spurious, noisy events. Events were then ascribed a unique identifier summarised using the regular expression “chr:strand:CasseteExon_start:CassetteExon_end:UpstreamExon_start:UpstreamExon_end:DownstreamExon_start:DownstreamExon_end” where start and end are the coordinates of the exons in question. This eventID was subsequently used to intersect and derive commonly regulated events across multiple conditions. For all downstream analyses only the SE or cassette exon type events were.

**Splicing quality control and wobble analyses:** To assess whether the control siRNA treated RBPMS over-expression samples aligned to their equivalents in the earlier experiment (Figs 2,3) i.e. whether the events regulated by RBPMS over-expression were consistent, we compared raw/unfiltered rMATS outputs between RBPMS high and low samples across experiments; this analysis revealed ~66-76% overlap of mapped or detected events as described by their eventIDs. With post-filtering for junction counts and ΔPSI (15% cutoff), the overlap reduced to between 41 and 44% approximately. Further, we performed detailed noise versus signal and wobble analyses to determine the source of the discrepancies between experiments. This highlighted experimental noise in both datasets arising from technical variations (ranging from cell line cultures to stochastic gene fragment sampling during sequencing) and some differences in mapping the exon coordinates that define each event (~6-8%). However, we observed that both unfiltered (n=55,985) and filtered (n=1185) overlapping events largely followed the same regulation patterns in both experiments as far as RBPMS is concerned (Supplementary Fig 5D) with correlation coefficients of 0.71 and 0.94 respectively (pearson correlation). This underlined the core regulated splicing patterns across experiments which are robustly maintained. To establish the comparable adult tissue network, the control siRNA + RBPMS-low samples were compared again via rMATS with aorta transcriptome (the overlap RBPMS – regulated network was largely retained in both experiments). We first extracted a set of all regulated and unregulated splicing events (n=32,561) that could be detected in adult aorta tissue and our four hESC-VSMC data-sets: RBPMS over-expressing control siRNA-treated (Ctrl-high) and RBFOX2 depleted (siRBFOX-high) and basal control siRNA-treated (Ctrl-low) and RBFOX2-depleted (siRBFOX-low). To delineate the RBFOX2 splicing regulatory network from these events – both individual and co-ordinated with RBPMS, we classified them into categories based on the splicing activity of RBPMS and RBFOX2 – whether included, skipped or unregulated. The final core set of 28 events within the adult aorta VSMC splicing network that were co-ordinately regulated by RBPMS and RBFOX2 are observed in both experiments 1 and 2.

**Gene Ontology analyses of alternatively spliced events:** For gene ontology analyses, genes containing alternatively spliced exons of the cassette exon (SE) category were used. Typically, events passing the 15% ΔPSI filter were considered except for the RBPMS high versus low comparisons in Supplementary Fig 4, where genes containing events passing a ΔPSI threshold of 30% were used to reduce the size of the network. GOprofiler was used with a custom background of all expressed genes in hESC-VSMCs with FDR filter of 0.05. For protein-protein network analyses, stringDB (<https://string-db.org/> version 11.0b)[21] was used with a whole genome background gene set. Typically, the terms presented were filtered for enrichment strength (Log10(observed / expected) threshold of 0.1 or more and false discovery rate of 0.01 or less (as specified in figure legends). In all analyses, the data set used were the high confidence (0.75 or more – specified in figure legends). Also, redundant terms containing the same sub-group of genes or broad, non-specific terms were excluded and only the most representative or encompassing GO terms are presented to showcase as many subgroups of genes as possible within these networks.

**Motif analyses:** Motif and exon-intron feature analyses of RBPMS-responsive alternatively spliced exons was performed using Matt [22] (v1.2.0). For these analyses only the skipped exon category events (SE outputs from rMATS), being by far, the large majority, were used. The motif corresponding to RBPMS binding sites are CAC clusters defined as CACN(1-12)CAC and the motif corresponding to RBFOX was defined as GCAC/TG. Specified motif searches were performed on the RBPMS-regulated exons and 250nt of the flanking introns using the rna_maps or the test_regexp_enrich tool. Test exons (inclusion/upregulated events and skipped/downregulated events) passed a threshold of FDR<0.05 and Inclusion Level Difference of 15% while background exons (Unregulated) were selected as events with FDR >0.1 and Inclusion level difference of <5%. For running rna maps, the background set was downsampled to a random 2000 events.

For an agnostic approach to identify putative splicing regulatory elements, kmer analysis specifically 8-mer analysis was performed using the test_regexp_enrich tool. Enriched 6mers and 8mers were identified for each comparison, separately for the following variants, following the structure of the Matt pipeline: upstream intron / exon / downstream intron, up-regulation / down-regulation, enrichment / depletion, first / internal / end. All possible 3mers, 4mers, and 5mers were then aligned to the identified enriched 6mers or 8mers to identify recurring short patterns with high enrichment scores for each condition. This analysis was performed in R, utilising the msa package (v1.24.0). The results were summarised using sequence logos visualisation, on information content, as well as heatmaps of the consensus sequences found. The colour gradient of each letter is proportional to the total observed enrichment.

**Correlation analyses:** To calculate the correlation between the expressions of RBPMS and MYH11 within adult SMCs, raw matrices for single cell RNA-seq data were downloaded from the adult heart cell atlas version 1 [23]. SMCs were isolated from the dataset using the annotations "SMC2_art" (n = 3,128), and "SMC1_basic" (n = 13,114), and both RBPMS and MYH11 were normalised by library sizes to give their expression per 10,000 UMIs, followed by a log transformation after adding a pseudocount of 1. The Spearman's rank correlation was calculated in the statistical computing environment R using cor.test(), and the line of best fit using the linear model function lm().

**Vector graphics and images:** were made using Adobe illustrator and Biorender (Biorender.com).

**References:**

1. Bertero, A., et al., *Optimized inducible shRNA and CRISPR/Cas9 platforms for in vitro studies of human development using hPSCs.* Development, 2016. **143**(23): p. 4405-4418.

2. Bertero, A., et al., *Conditional Manipulation of Gene Function in Human Cells with Optimized Inducible shRNA.* Curr Protoc Stem Cell Biol, 2018. **44**: p. 5C 4 1-5C 4 48.

3. Pawlowski, M., et al., *Inducible and Deterministic Forward Programming of Human Pluripotent Stem Cells into Neurons, Skeletal Myocytes, and Oligodendrocytes.* Stem Cell Reports, 2017. **8**(4): p. 803-812.

4. Nakagaki-Silva, E.E., et al., *Identification of RBPMS as a mammalian smooth muscle master splicing regulator via proximity of its gene with super-enhancers.* Elife, 2019. **8**.

5. Cheung, C., et al., *Directed differentiation of embryonic origin-specific vascular smooth muscle subtypes from human pluripotent stem cells.* Nat Protoc, 2014. **9**(4): p. 929-38.

6. Davaapil, H., et al., *A phenotypic screen of Marfan syndrome iPSC-derived vascular smooth muscle cells uncovers GSK3beta as a new target.* Stem Cell Reports, 2023. **18**(2): p. 555-569.

7. Granata, A., et al., *An iPSC-derived vascular model of Marfan syndrome identifies key mediators of smooth muscle cell death.* Nat Genet, 2017. **49**(1): p. 97-109.

8. Holt, P.J., et al., *Differentiation and quality control of smooth muscle cells from human pluripotent stem cells via the neural crest lineage.* bioRxiv, 2023: p. 2023.05.31.543049.

9. Williams, E.C., et al., *The Sum of Two Halves May Be Different from the Whole-Effects of Splitting Sequencing Samples Across Lanes.* Genes (Basel), 2022. **13**(12).

10. Dobin, A., et al., *STAR: ultrafast universal RNA-seq aligner.* Bioinformatics, 2013. **29**(1): p. 15-21.

11. Mohorianu, I., et al., *Genomic responses to the socio-sexual environment in male Drosophila melanogaster exposed to conspecific rivals.* RNA, 2017. **23**(7): p. 1048-1059.

12. Moutsopoulos, I., et al., *noisyR: enhancing biological signal in sequencing datasets by characterizing random technical noise.* Nucleic Acids Res, 2021. **49**(14): p. e83.

13. Bolstad, B.M., et al., *A comparison of normalization methods for high density oligonucleotide array data based on variance and bias.* Bioinformatics, 2003. **19**(2): p. 185-93.

14. Robinson, M.D., D.J. McCarthy, and G.K. Smyth, *edgeR: a Bioconductor package for differential expression analysis of digital gene expression data.* Bioinformatics, 2010. **26**(1): p. 139-40.

15. Raudvere, U., et al., *g:Profiler: a web server for functional enrichment analysis and conversions of gene lists (2019 update).* Nucleic Acids Res, 2019. **47**(W1): p. W191-W198.

16. Kanehisa, M. and S. Goto, *KEGG: kyoto encyclopedia of genes and genomes.* Nucleic Acids Res, 2000. **28**(1): p. 27-30.

17. Viteri, G., et al., *Reactome and ORCID-fine-grained credit attribution for community curation.* Database (Oxford), 2019. **2019**.

18. Moutsopoulos, I., E.C. Williams, and I.I. Mohorianu, *bulkAnalyseR: An accessible, interactive pipeline for analysing and sharing bulk multi-modal sequencing data.* bioRxiv, 2022: p. 2021.12.23.473982.

19. Shen, S., et al., *rMATS: robust and flexible detection of differential alternative splicing from replicate RNA-Seq data.* Proc Natl Acad Sci U S A, 2014. **111**(51): p. E5593-601.

20. Zhou, X., et al., *Transcriptome and N6-Methyladenosine RNA Methylome Analyses in Aortic Dissection and Normal Human Aorta.* Front Cardiovasc Med, 2021. **8**: p. 627380.

21. Szklarczyk, D., et al., *The STRING database in 2021: customizable protein-protein networks, and functional characterization of user-uploaded gene/measurement sets.* Nucleic Acids Res, 2021. **49**(D1): p. D605-D612.

22. Gohr, A. and M. Irimia, *Matt: Unix tools for alternative splicing analysis.* Bioinformatics, 2019. **35**(1): p. 130-132.

23. Litvinukova, M., et al., *Cells of the adult human heart.* Nature, 2020. **588**(7838): p. 466-472.
