# Supplementary material for: RNA binding protein with multiple splicing (RBPMS) promotes contractile phenotype splicing in human embryonic stem cell–derived vascular smooth muscle cells
Source: Cardiovasc Res. 2024 Sep 9;120(16):2104–16. doi: 10.1093/cvr/cvae198 (PMC11646123; doi:10.1093/cvr/cvae198)
Supplement: cvae198_Supplementary_Data [file cvae198_supplementary_data.zip › Supplementary Fig legends_V12.2_clean.docx]

**Supplementary Fig 1.** GTEX bulk human adult tissue mRNA-seq IGV tracks showing expression of SM-exons (arrow) in **A.** *ITGA7* and **B.** *MYOCD* in VSMC rich artery tissues – aorta, coronary and tibial (green) and in non-smooth muscle i.e. striated muscle tissues – heart (left and right ventricles) and skeletal muscle (blue). *ITGA7* exon 5 is one of a mutually exclusive pair; exon 6 is used in cardiac and skeletal muscle.

**Supplementary Fig 2. A.** *RBPMS* transcripts are expressed at detectable levels in neural crest derived hESC-VSMCs. Shown are quantitative RT-PCR data – Ct (amplification cycle) values of *RBPMS* and *GAPDH* in VEC and RBPMS-hESC-VSMCs lines (N=4, derived from 2 independent VEC-hESC and 2 independent RBPMS hESC clonal lines from multiple neural crest differentiations). These are endogenous *RBPMS* levels without Doxycycline induction. **B.** Immunoblots showing RBPMS protein levels in VSMCs, differentiated via the neural crest (NC – n=4) and the lateral plate mesoderm (LM – n=2) lineages compared to H9 human embryonic stem cells and NC or LM. GAPDH is the loading control. The schematic shows the differentiation timeline. The bar graph shows quantification of RBPMS expression relative to GAPDH (and normalised to H9 human ESCs) in NC derived hESC-VSMCs across n=4 immunoblots. The number of biological replicates of the individual samples is indicated and also represented as different symbols on the graph. **C-E.** Data are from the human heart cell atlas portal - vascular data [1]. **C.** UMAPS of the cells classified as smooth muscle show 3 main clusters consisting of SMC-art (arterial) and SMC-basic cells that are considered more contractile and more proliferative respectively. Colour scales indicate relative gene expression levels of RBPMS and MYH11 in these cells. Generated using visualization tool CellXgene of the “vascular” data from the human heart cell atlas portal. **D.** RBPMS correlates with MYH11 expression in vascular smooth muscle cells with Spearman’s Rho = 0.24, p < 0.001. Shown is a correlation scatter plot of cells classified as SMCs (arterial and basic) with the axes showing gene expression of *RBPMS* or *MYH11* as log counts per 10,000 UMIs. Colour scales indicate relative cell density within each quadrant – RBPMS and MYH11 double positive (45%), RBPMS positive MYH11 negative (8%), RBPMS negative MYH11 positive (28%) and double negative. **E.** The heatmap matrix shows the correlation coefficients of MYH11 with RBPMS and other VSMC markers like CNN1, TAGLN, ACTA2. It is worth noting that while the Rho value of RBPMS correlation with MYH11 is lower than those of the VSMC markers, it is still positively correlated, statistically significant and is of the same order of magnitude as the key VSMC transcriptional master regulator MYOCD. TNNT2 (cardiac), PECAM1 (endothelial cell), UPK3B (Epicardial cells) and GAPDH (housekeeping gene) show 0 or negative correlation with MYH11. Colour scale bar shows Spearman’s Rho and the values are also indicated in the matrix.

**Supplementary Fig 3. A.** Doxycycline treated, lateral plate mesoderm derived Vec and RBPMS VSMCs were sorted for GFP intensity using flow cytometry and the fractions were collected and RNA was isolated. RT-PCR of selected SM-AS targets from the Doxycycline-treated low, medium and high GFP VSMC fractions of the Vec and the RBPMS cells. All targets tested showed statistically significant RBPMS dose responsive induction of the SM splicing event. “Percent” of the SM isoform is graphed (as in Fig 1). N=3 for the RBPMS line and N=2 for the Vec line where each bioreplicate represents an independent differentiation experiment. RBPMS lines were derived from 2 independent clones. Note that in some experiments in the Vec line where GFP intensity is generally higher than the RBPMS line, only the high GFP intensity cells could be collected for RNA isolation. Hence statistical analysis was not performed in the VEC samples although the RT-PCR results were reproducible. SM exon inclusion was compared between RBPMS VSMCs of low, mid or high GFP gates using 2-WAY ANOVA multiple comparisons without correction (Fisher’s LSD). Pvalues <0.01 **, < 0.05 *. Only statistically significant comparisons are indicated. RBPMS-GFP low-neg vs. RBPMS-GFP mid: MYOCD=0.0432, SMTN=0.0307, CALD1=0.0355, ACTN1=0.0345, RBPMS-GFP low-neg vs. RBPMS-GFP high: MYOCD=0.0362, SMTN=0.0092, VCL=0.0360, CALD1=0.0066, ACTN1=0.0307, RBPMS-GFP mid vs. RBPMS-GFP high: SMTN=0.0.0036, VCL=0.0156, CALD1=0.0042. **B.** Sashimi tracks showing *SMTN* and *TPM1* SM isoform (arrow) induction by RBPMS over-expression in a manner similar to adult aorta tissue (obtained from GSE147026) [2]. PSI values are shown with +/- standard deviation (SD) across N=3 replicates as predicted by rMATS. Note: For *SMTN***,** skipping of the non SM exon (arrow) in the SM isoform is represented as decreasing PSI values and is seen by the increase in skipped exon junction reads exclusive to the +Dox and adult aorta samples.  **C.** Lysates from neural crest derived RBPMS-hESC-VSMCs either untreated or treated with 0.2ug/ml Doxycycline were separated on a 10% polyacrylamide gel and blotted and probed for Vinculin (VCL) expression. RBPMS over-expression shows induction of the meta-VCL protein isoform matching transcript expression of meta-*VCL* - RT-PCR and bulk mRNA sequencing data. **D.** Graph showing quantile normalised gene expression of *RBPMS* from bulk mRNA sequencing of RBPMS-hESC-VSMCs (no Dox, low and high, N=4) and adult human aorta tissue [2]. Statistical significance was tested using one-way ANOVA with Šídák's multiple comparisons test.

**Supplementary Fig 4. A.** K-mer logo analysis shows enriched 5-mers on and flanking the RBPMS-regulated exons. 8-mer analysis using MATT of the RBPMS regulated exons (cassette exon category from rMATS) and 250bp of the flanking introns represented as motif logos. Test and background exons were designated as in Fig 5A. Heat maps depict enrichment scores of the various k-mers at each location on and flanking the regulated exons. **B.** RNA map showing that disruption of the optimal RBFOX2 motif in the linked Perl expression results in a loss of enrichment suggesting that the co-occurrence of RBPMS binding motifs with RBFOX2 is specific.

**Supplementary Fig 5. A.** Heat map of TPM (transcripts per million) values showing expression of the RBFOX family members in the hESC-VSMCs (H9 rTTA NC-VSMC - experiment 2 control samples), adult aorta tissue (GSE147026) and adult arterial tissues – tibial, coronary and aorta from GTEX. **B.** RBFOX2 knock-down experiment with either targeted or control non-specific siRNA treatment of hESC-VSMCs cultured with or without 0.2ug/ml Doxycycline. Data are shown for hESC-VSMCs derived via neural crest lineage from 1 vector control (Vec) hESC line and 2 RBPMS hESC clones. Each differentiation was performed once. Electropherograms show RT-PCRs of selected targets *ACTN1* and *SMTN* for the SM exons/isoforms. Quantitated Percent Spliced In (PSI) shown below. RBFOX2 depletion substantially reverted the RBPMS-induction of SM exon in *ACTN1* and *SMTN*. **C.** Immunoblotting (right) showing knockdown of RBFOX2 in hESC-VSMCs. GAPDH is used as a loading control. **D.** Immunoblotting for RBFOX2 and GAPDH (loading control) for the bulk mRNA sequencing experiment described in Fig 3C. **E.** Bar plot showing quantile normalised gene expression values of RBFOX2 in hESC-VSMCs (with or without anti-RBFOX2 siRNA) and adult aorta (N=3). Statistical significance was tested using one-way ANOVA with Šídák's multiple comparisons test. **F.** Cross plots showing the high correlation (pearson) in splicing patterns in the common events between experiments 1 and 2 for both unfiltered and ΔPSI 15% filtered outputs in RBPMS – low and high conditions.

**Supplementary Fig 6. Gene Ontology and protein-protein network analyses of the RBPMS –regulated cassette exons shows functionally critical gene networks for VSMCs. A.** GOprofiler analyses of the RBPMS-regulated genes (RStudio) using custom background gene sets (all expressed genes in hESC-VSMCs). Enriched terms include actin cytoskeleton and focal adhesion components across the Cellular Component (CC), Molecular Function (MF) and Biological Process (BP) categories. Regulated exons were defined as those showing >= 30% splicing differences and FDR <0.05 (Benjamini-Hochberg) **B.** Protein-protein interaction network analysis with StringDB (string-db.org) (whole genome background). Top 15 GO terms from the categories – Cellular Component, Molecular Function and Biological Process are shown, arranged by –log10 FDR values (high to low) are shown. These were filtered limiting to those terms that contained at least 25 genes in the background set in that category and had a strength (log 10 observed/expected) of 0.3 or more.

**Supplementary Fig 7. A.** Genes showing significant alternative splicing (ΔPSI >=15% and FDR < 0.05) between adult human aorta and hESC-VSMCs (RBPMS-low) representing the SM-AS events, were examined for protein-protein interaction networks with StringDB (string-db.org). Whole genome background was used for the analyses. Dot plots showing top enhanced enriched terms in the Molecular Function, Cellular Component and Biological Process categories respectively connecting high confidence nodes of interaction scores 0.99 or more. The terms enriched are represented with –log10 FDR and filtered from StringDB analyses for term strength (log 10 observed/expected) of 0.25 or more and FDR <= 0.001. SM-AS events are enriched for terms associated with the actin cytoskeleton and focal adhesion components (marked with arrow) similar to the RBPMS-regulated network indicating functional significance for VSMCs. **B.** rMATS analysis comparing siRBFOX2 treated samples with cognate controls were mapped back on to the adult aorta splicing network (rMATS comparison of adult aorta vs Control-siRNA RBPMS-low hESC-VSMCs) and events were classified based on regulation by RBPMS and RBFOX2 of the adult aorta tissue network. Box plots summarize the 28 core events where RBPMS and RBFOX2 co-ordinately contribute to the adult tissue VSMC splicing programme. Number of events falling into each category after all filters were set, are indicated. Adult_incl = higher exon inclusion in adult aorta and Adult_skip = higher exon skipping in adult aorta. Events classified as regulated – included or skipped showed ΔPSI >= 15% and FDR < 0.05. Average PSIs across 3 biological replicates are plotted. **C.** Box plots showing the Percent Spliced In (PSI) distribution of the RBPMS-responsive non-SM-AS clusters 3 and 4 from the heatmap in 4A. In these events RBPMS regulation induces splicing patterns divergent from those seen in adult aorta tissue.

**Supplementary Fig 8.** Motility of GFP high and low cells of 2 independent Vector control hESC-VSMC lines are shown. 40 cells for clone 1 and 38 cells for clone 2 across 2 wells were tracked in each category. GFP high and low cells do not show significant differences in their motility (2-way ANOVA multiple comparison without correction independent Fishers LSD-tests).

**Uncropped images.** All immunoblots and cropped RT-PCR capillary electrophoresis images are shown as uncropped versions with appropriate size markers included. Lanes used are labelled and indicated by boxes (Fig 1A).

**Supplemental tables:**

**Supplemental table 1:** List of all AS events in – A. Experiment_1 = RBPMS high versus low comparisons in experiment 1, B. Adult_RBPMS_low.Experiment_1 = the comparison of adult aorta tissue and basal hES-VSMC events (RBPMS low conditions) in experiment 1, C. Adult_RBPMS_core_events_F4_assoc = the clusters of AS events associated with Fig 4 comparing the RBPMS-driven network in hES-VSMCs with adult aorta splicing, D. Experiment_2_Adult_data = AS events in experiment 2 with RBFOX2 depletion induced events in comparison with the adult aorta splicing network. PSI = Percent Spliced In, Event_ID is described in the methods section and denotes the genomic coordinates of the alternatively spliced event and flanking junctions.

**Supplemental table 2:** Results of the Matt k-mer (6-mer) enrichment analysis on the flanking introns and the alternatively spliced cassette exons regulated by RBPMS from experiment 1 are shown. Table lists enrichment scores and pValues as generated by the test_regexp_enrich tool with 1000 iterations. The test set represents all significantly (FDR<0.05) included (up_DIFF – 1012 sequences) or skipped (down_DIFF – 1690 sequences) exons with at least 15% ΔPSI and the background set represents unregulated exons (2000 sequences). 1000 permutations were performed and pvalues and enrichment scores are shown for the entire (ALL) intron or exon and also divided into 3 bins - first, internal and end region (for the introns going proximal to distal from the exon in the middle). Number of hits (NHITS) for each motif in the sequences is also presented. Typically, pvalue < 0.001 i.e. ** was considered to be a significant enrichment and those 6-mers were further used for logo analysis.

**Supplemental table 3:** Gene ontology of RBPMS-adult spliced network events listing StringDB outputs of genes in clusters 1 and 2 (RBPMS-driven adult network) and those in clusters 3 and 4 listed in Supplemental table 1.

1. Litvinukova, M., et al., *Cells of the adult human heart.* Nature, 2020. **588**(7838): p. 466-472.

2. Zhou, X., et al., *Transcriptome and N6-Methyladenosine RNA Methylome Analyses in Aortic Dissection and Normal Human Aorta.* Front Cardiovasc Med, 2021. **8**: p. 627380.
