# Supplementary figures and images for: RNA binding protein with multiple splicing (RBPMS) promotes contractile phenotype splicing in human embryonic stem cell–derived vascular smooth muscle cells
Source: Cardiovasc Res. 2024 Sep 9;120(16):2104–16. doi: 10.1093/cvr/cvae198 (PMC11646123; doi:10.1093/cvr/cvae198)

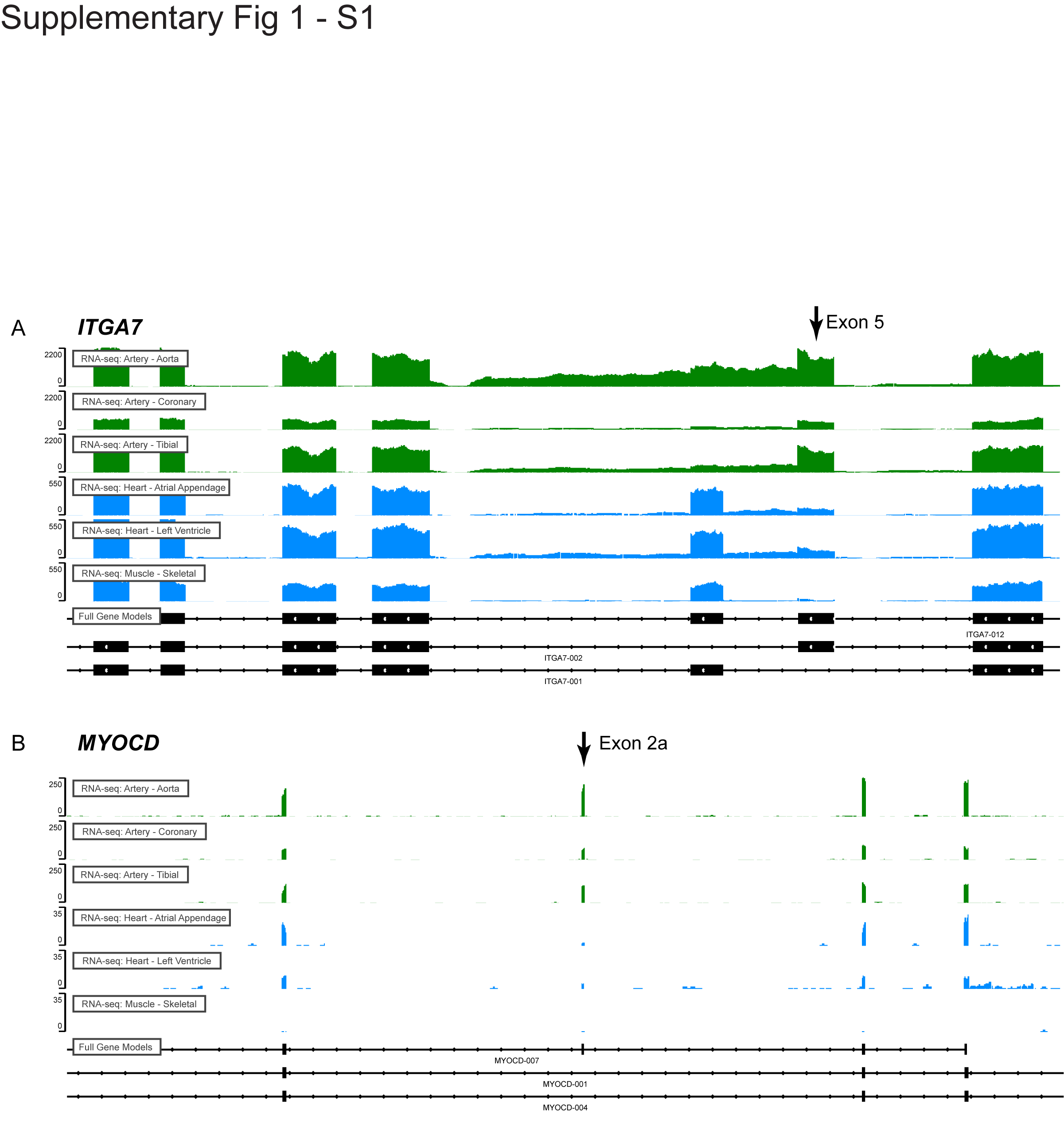

Supplement: cvae198_Supplementary_Data [file cvae198_supplementary_data.zip › Fig1_Supp_associated_1_S1.tif]

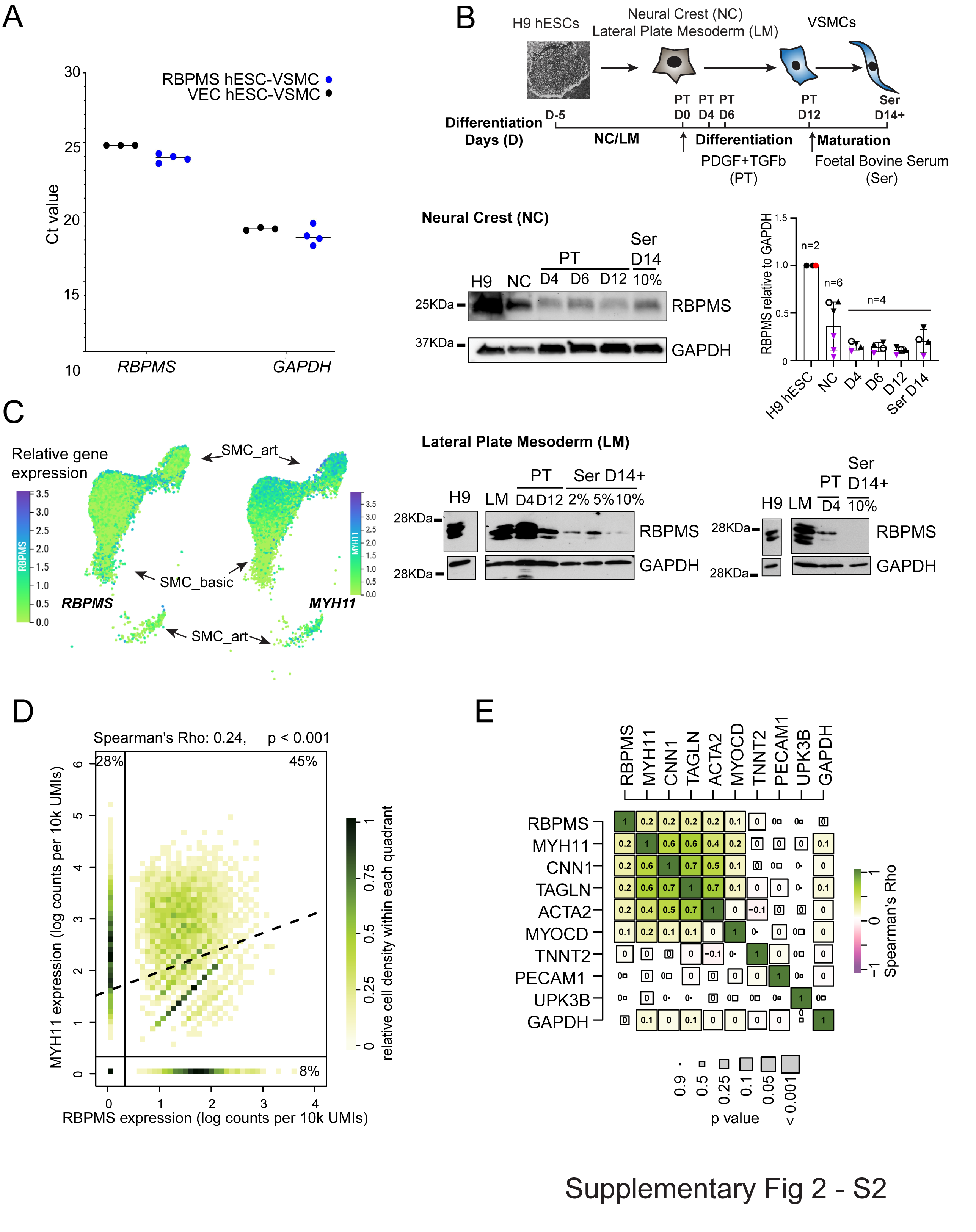

Supplement: cvae198_Supplementary_Data [file cvae198_supplementary_data.zip › Fig1_Supp_associated_2_S2_ver7.tif]

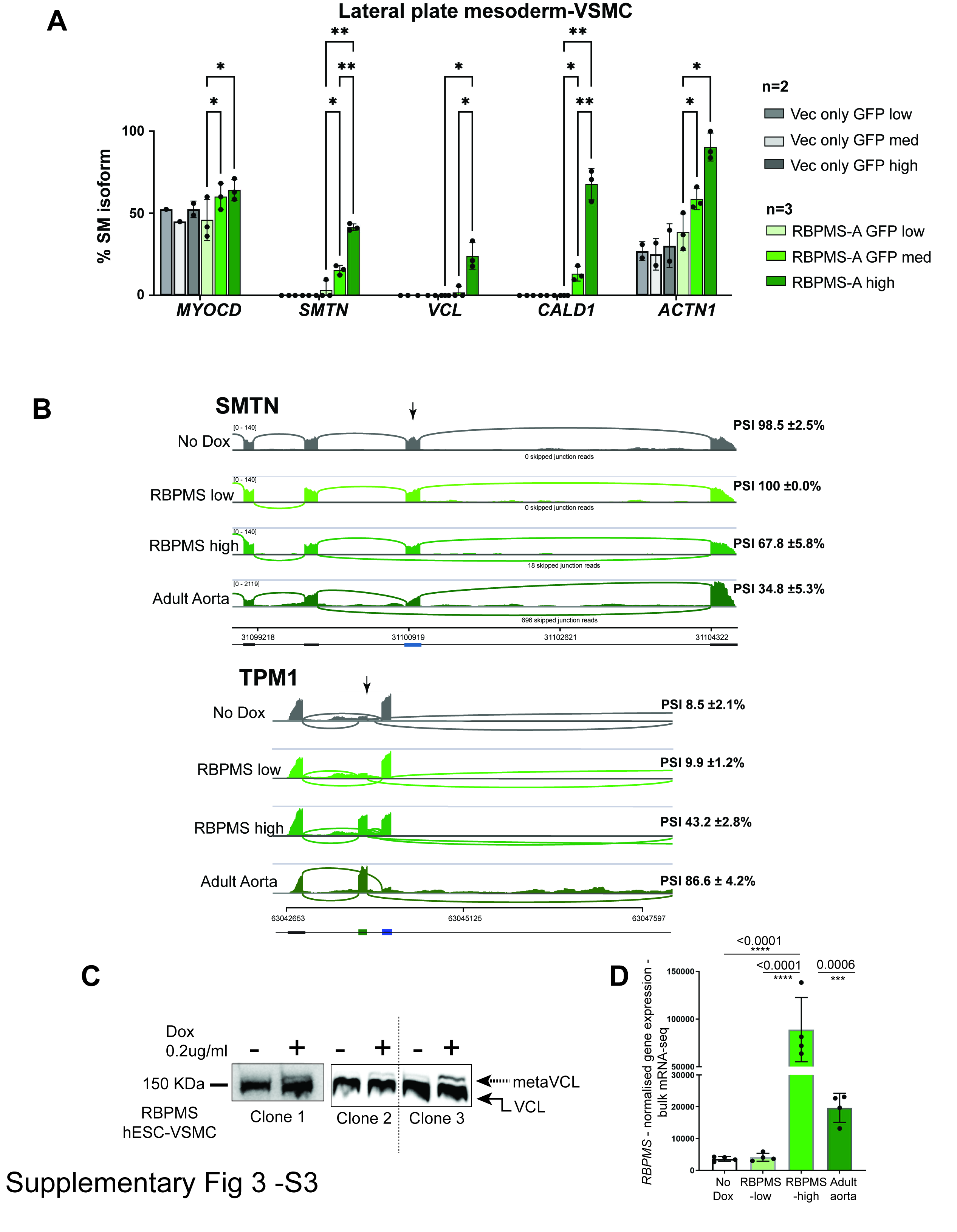

Supplement: cvae198_Supplementary_Data [file cvae198_supplementary_data.zip › Fig2_Supp_associated_1_S3.tif]

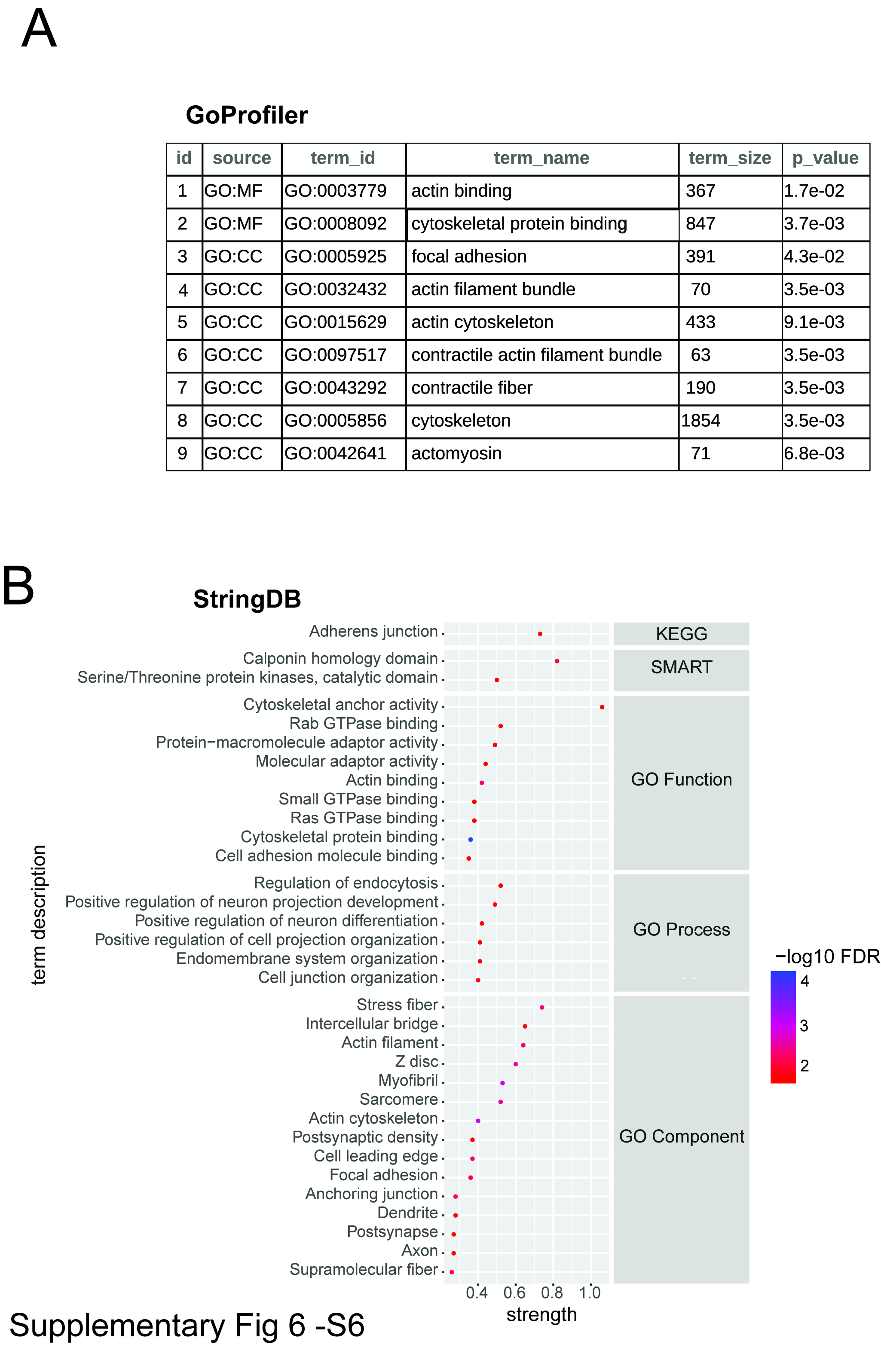

Supplement: cvae198_Supplementary_Data [file cvae198_supplementary_data.zip › Fig2_Supp_associated_3_S6.tif]

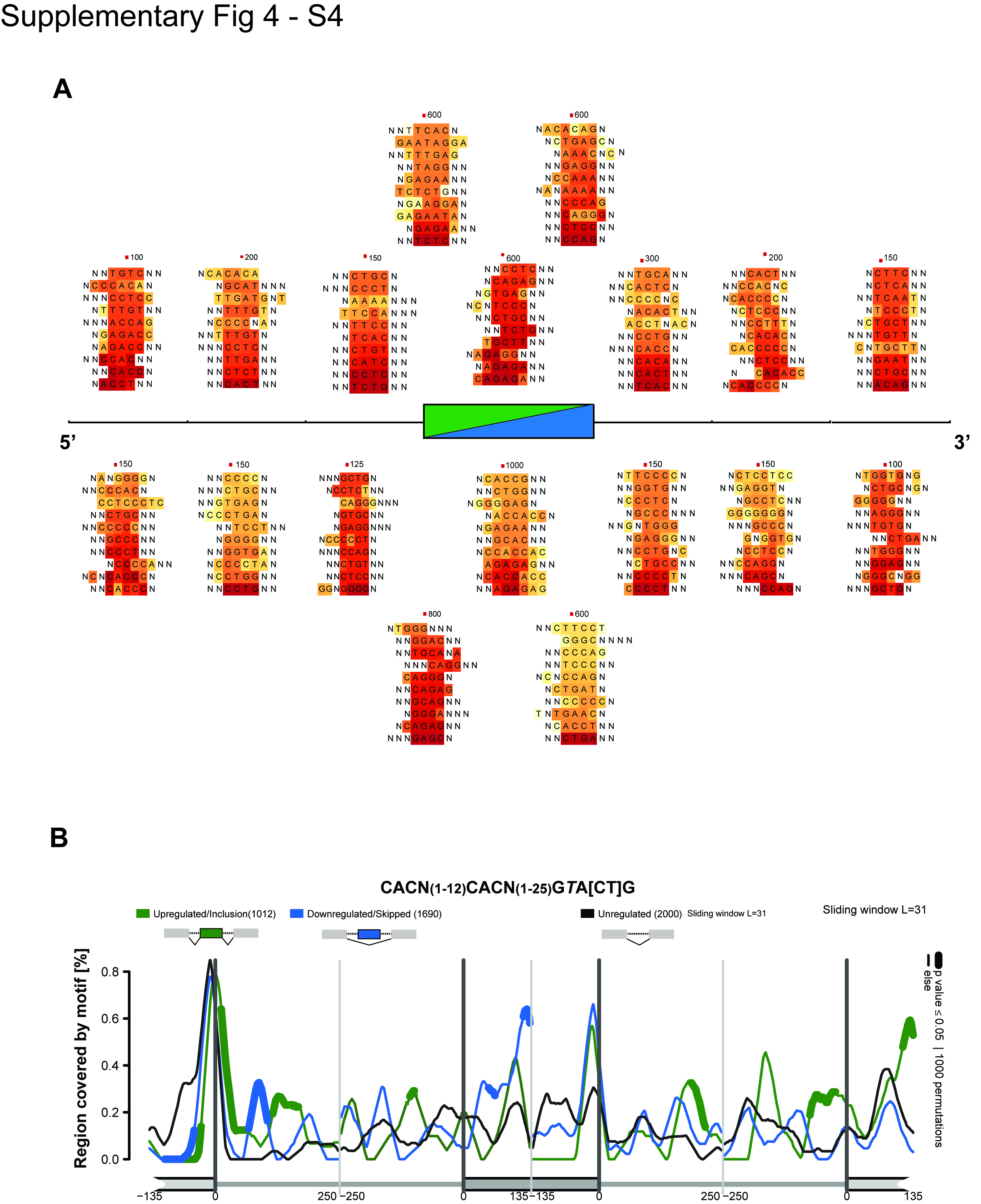

Supplement: cvae198_Supplementary_Data [file cvae198_supplementary_data.zip › Fig3_Supp_associated_1_S4.tif]

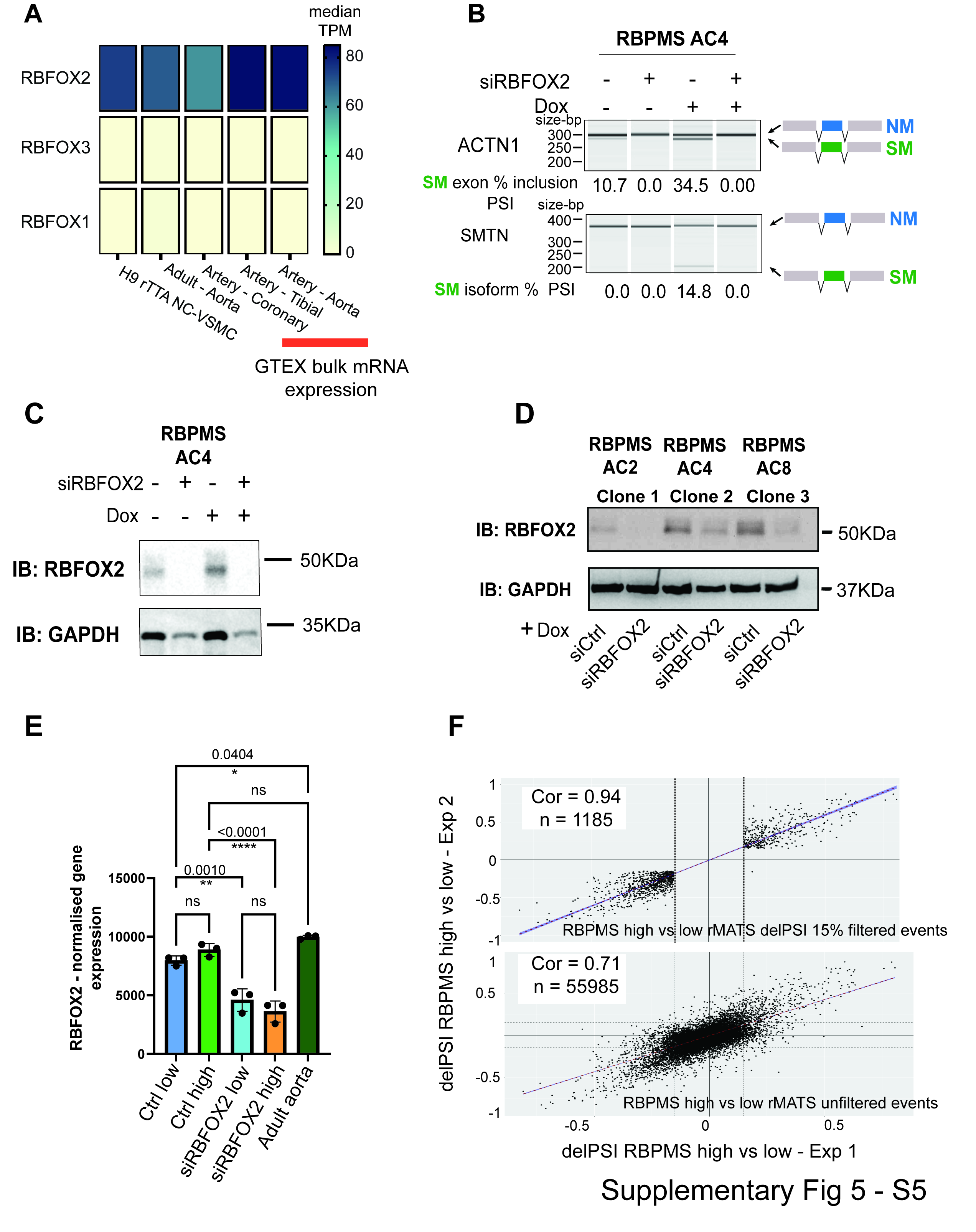

Supplement: cvae198_Supplementary_Data [file cvae198_supplementary_data.zip › Fig3_Supp_associated_2_S5_ver2.tif]

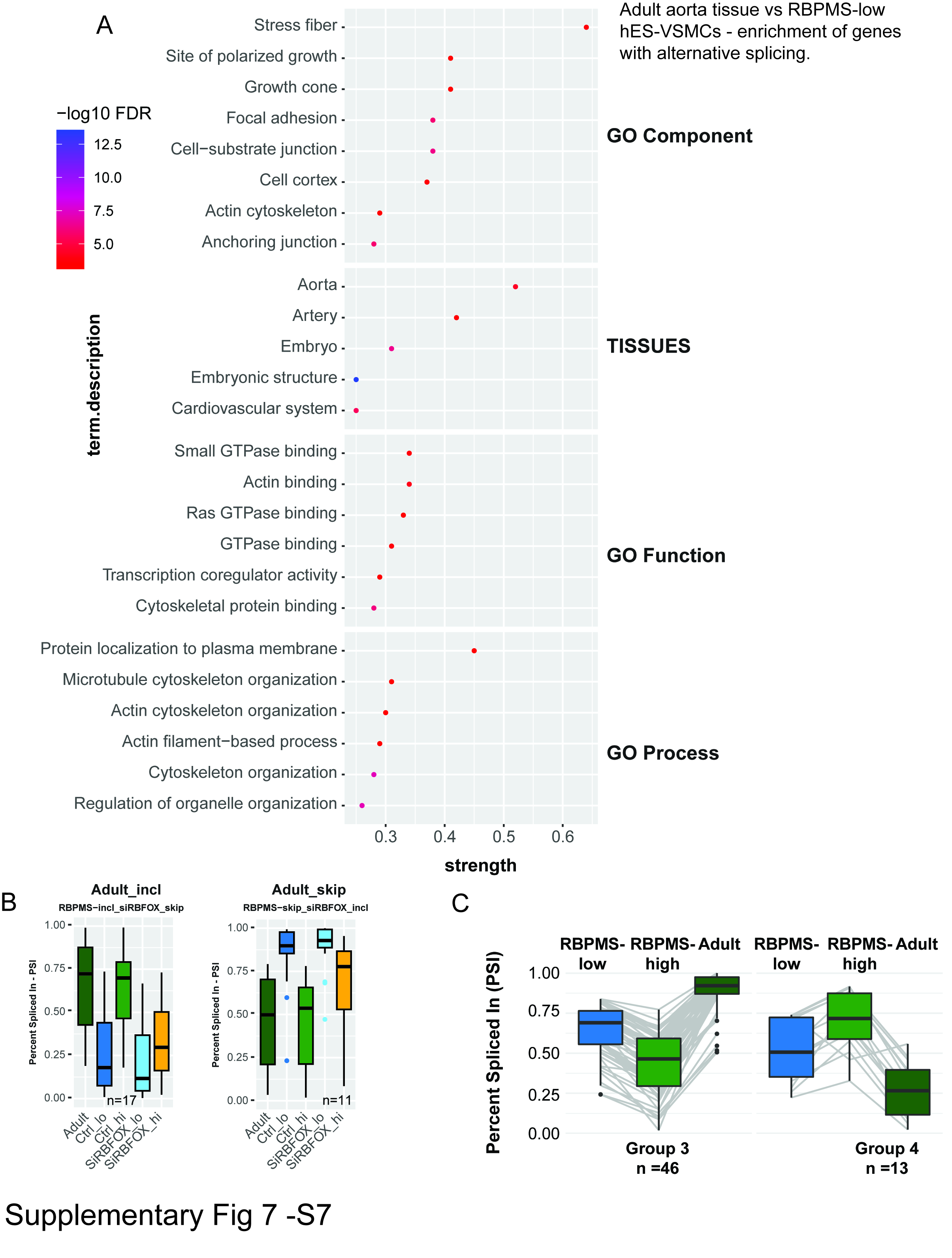

Supplement: cvae198_Supplementary_Data [file cvae198_supplementary_data.zip › Fig4_Supp_associated_S7.tif]

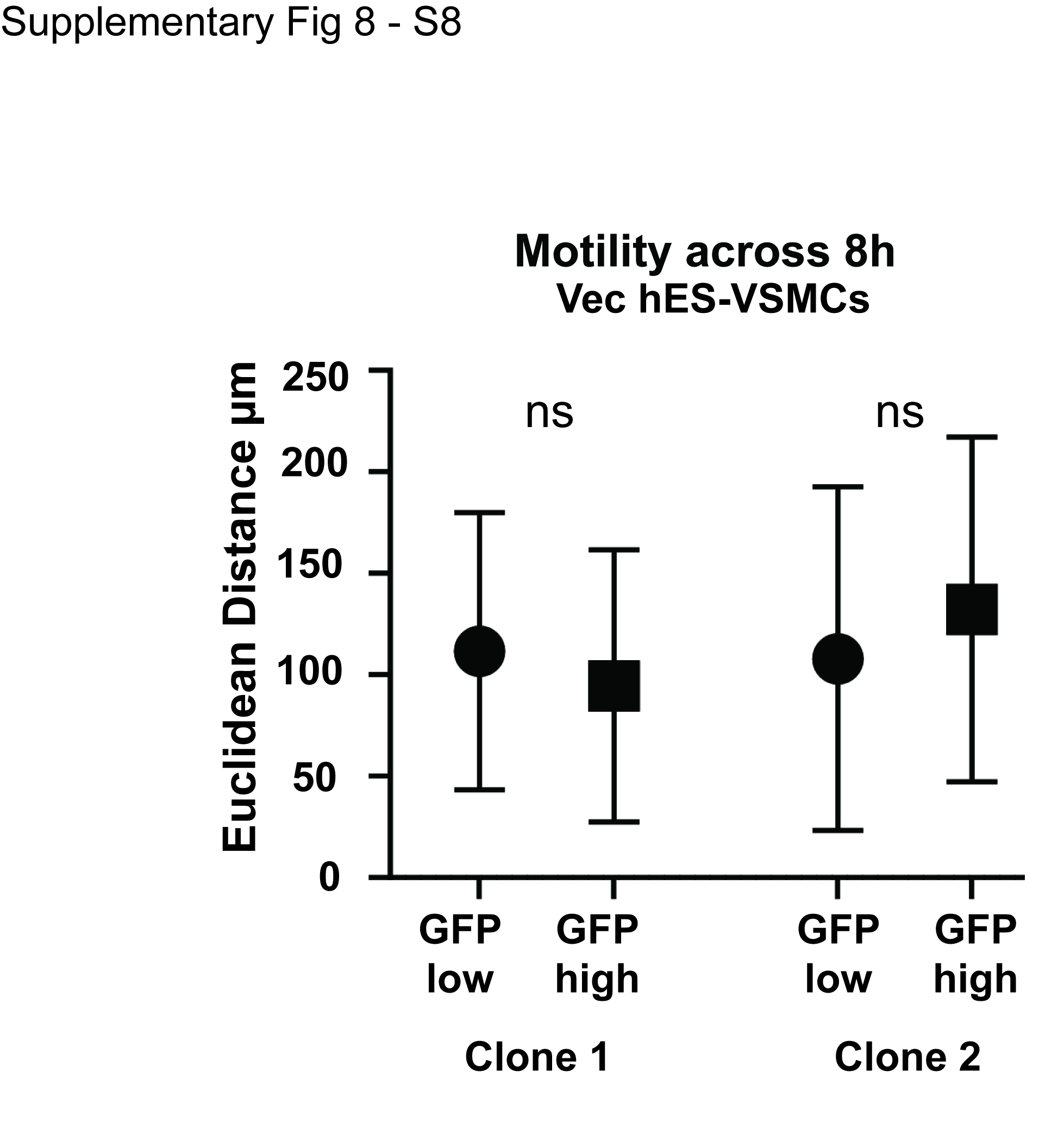

Supplement: cvae198_Supplementary_Data [file cvae198_supplementary_data.zip › Fig5_Supp_associated_S8.tif]
